# Flow Cytometric and 16S Sequencing Methodologies for Monitoring the Physiological Status of the Microbiome in Powdered Infant Formula Production

**DOI:** 10.3389/fmicb.2016.00968

**Published:** 2016-06-22

**Authors:** Amir H. P. Anvarian, Yu Cao, Shabarinath Srikumar, Séamus Fanning, Kieran Jordan

**Affiliations:** ^1^Teagasc, Food Research CentreFermoy, Ireland; ^2^UCD Centre for Food Safety, Science Centre South, University College DublinDublin, Ireland

**Keywords:** powdered infant formula (PIF), systems microbiology, flow cytometry, 16S sequencing, viable but non-culturable (VBNC), microbial stress response, environmental sampling, microbial physiology

## Abstract

The aim of this study was to develop appropriate protocols for flow cytometric (FCM) and 16S rDNA sequencing investigation of the microbiome in a powdered infant formula (PIF) production facility. Twenty swabs were collected from each of the three care zones of a PIF production facility and used for preparing composite samples. For FCM studies, the swabs were washed in 200 mL phosphate buffer saline (PBS). The cells were harvested by three-step centrifugation followed by a single stage filtration. Cells were dispersed in fresh PBS and analyzed with a flow cytometer for membrane integrity, metabolic activity, respiratory activity and Gram characteristics of the microbiome using various fluorophores. The samples were also plated on agar plates to determine the number of culturable cells. For 16S rDNA sequencing studies, the cells were harvested by centrifugation only. Genomic DNA was extracted using a chloroform-based method and used for 16S rDNA sequencing studies. Compared to the dry low and high care zones, the wet medium care zone contained a greater number of viable, culturable, and metabolically active cells. Viable but non-culturable cells were also detected in dry-care zones. In total, 243 genera were detected in the facility of which 42 were found in all three care zones. The greatest diversity in the microbiome was observed in low care. The genera present in low, medium and high care were mostly associated with soil, water, and humans, respectively. The most prevalent genera in low, medium and high care were *Pseudomonas, Acinetobacter*, and *Streptococcus*, respectively. The integration of FCM and metagenomic data provided further information on the density of different species in the facility.

## Introduction

Currently, culture-based methods such as agar plates are the most commonly used technique for assessing the microbiological status of food processing environments. In using agar plates, it is only possible to determine the presence of bacteria that the investigator is looking for (using selective agars) and the method gives little information on the physiological status of the cells, except that they are alive if they grow. Some additional information can also be obtained by using different nutrient-content agars and assessing the number of stressed cells. With advances in microbiological techniques, there are a number of different methodologies that can be applied to samples to gain more information about the bacterial population of a processing environment and the physiological state of the bacteria present. Two of these methodologies are 16S rDNA sequencing and flow cytometry (FCM), the former giving information about the type of bacteria present and the latter providing information on the physiological state of the bacteria. Combining the results of these two methods can give valuable information about the microbiological status of a food processing environment.

Though most micro-organisms are cultured using traditional culture-based methods, under stress conditions, many microorganisms, some yet unidentified, are unable to grow on conventional growth media due to lack of effective culture techniques and/or induction of the so-called viable but non-culturable (VBNC) state (see Quigley et al., 2011; Ramamurthy et al., 2014). This has made normal culture techniques ineffective in describing the entire microbiome of complex environments. This problem could be overcome by using 16S rDNA sequencing, which is a culture-independent next generation sequencing method and has been successfully used for describing the composition of the microbiome in depth. The 16S rDNA gene encodes for 16S ribosomal RNA which is universally present in prokaryotic microorganisms (Coenye and Vandamme, 2003). The variations within the 16S rDNA sequences facilitate identification of bacteria at the species level and, therefore, has made 16S rDNA gene sequencing an ideal tool for bacterial taxonomic studies (Neefs et al., 1993). Even though 16S rDNA gene sequencing is widely used to identify inter-species variations, the development of modern high throughput sequencing technologies combined with downstream bioinformatics analysis has made this tool ideal for species identification within complex communities (Logares et al., 2014). It has been shown that 16S rDNA amplicon fragments as short as ~82 base pairs are sufficient for classification at the phylum level (Lazarevic et al., 2009), and with good primer design and analysis methods, fragments of 100–200 base pairs could show the same clustering information as long fragments used in phylogenetic studies (Liu et al., 2007). Compared to other molecular microbiology techniques, 16S rDNA sequencing is not only cheaper in price, but also the interpretation of the resulting data is easier and faster. With some online analysis platforms, such as Illumina Basespace or software such as QIIME and MOTHUR (Schloss et al., 2009; Caporaso et al., 2010) one could easily generate very straightforward information and give an overview about detailed structures and compositions of the target environment microbiome. However, such methodologies will detect gene sequences and will not differentiate live from dead cells.

Flow cytometry is a powerful and rapid technique for simultaneous quantification and multi-parameter analysis of the microbial populations at single cell level (Müller and Nebe-Von-Caron, 2010). Cells are focused and aligned one behind the other in a narrow stream with a diameter close to the diameter of the cells so that single cells can be introduced to the light beam (generally laser). When cells are subjected to the light, they scatter light in all directions, although it is generally detected in two directions: forward scatter (FSC), along the axis of the light source; and side scatter (SSC), perpendicular to the light beam. Data from FSC and SSC scatters are generally used to characterize the morphological state of the cells, as rough indicators of the cell size and granularity, respectively. In addition, the light absorbed by the cells can result in emission of fluorescence (either due to presence of naturally fluorescent compounds or staining with various fluorophores), the intensity of which could be detected by FCM (Shapiro, 2003). Consequently, staining the cells with various fluorophores or fluorescence-conjugated antibodies can be used for understanding a wide range of physiological parameters of the cell (e.g., viability, metabolic and respiratory activities, internal pH, etc.) as well as detection of specific microorganisms at an analysis rate of up to 10,000 cells per second. Furthermore, comparing the viability results obtained with FCM with those of the plate counting can be used to determine the number of VBNC cells in a sample.

The aim of this study was to develop appropriate protocols for flow cytometry and 16S rDNA sequencing investigation of the environmental microbiome in a powdered infant formula (PIF) production facility in the Republic of Ireland.

## Materials and methods

### Sampling

**Figure 1:**
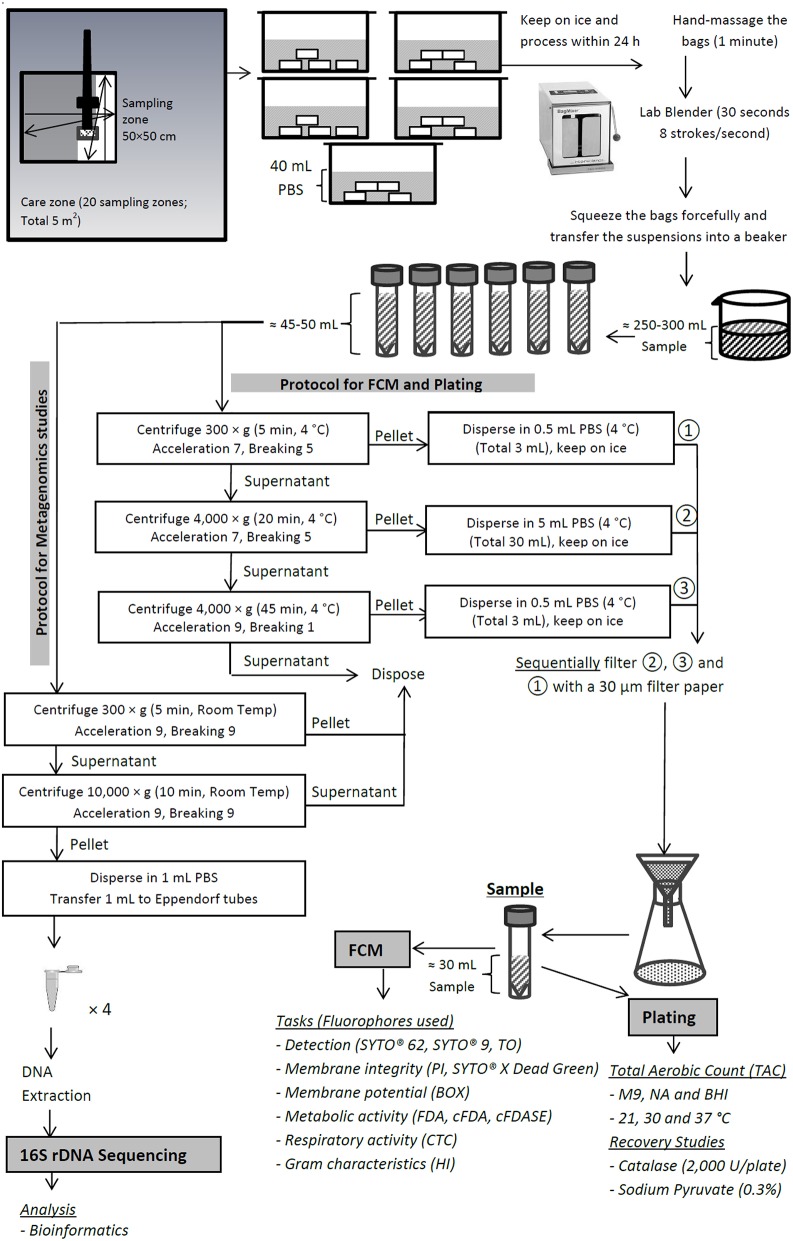
**Schematic representation of the steps involved in sampling and sample preparation for flow cytometric and 16S rDNA sequencing studies**.

The PIF production unit consisted of three care zones of low, medium and high care. From each zone, twenty swab samples were collected, representing the critical production, storage and packaging sites within each PIF production zone (see Supplementary Table 1). Sponges pre-moistened with neutralizing buffer (Labplas Inc., Sainte-Julie, Canada) were used for environmental swabbing. Each hydrated sponge was used for swiping a single sampling zone of 50 × 50 cm. The zigzag wiping procedure for surface sampling was performed as described by Nicolau and Bolocan (2014). In total, the twenty samples taken represented 5 m^2^ of that zone and were placed into five bags, each consisting of four sponges. To each bag, 40 mL of phosphate buffered saline (PBS; Sigma, St Louis, USA) was added not earlier than 30 min post-sampling. The purpose of the delay was to prevent the dilution of the neutralizing buffer, allowing the effective neutralization of the possible chlorine and quaternary ammonium compound residues in the sample. The bags were sealed with the tabs provided, kept on ice, transported to the laboratory and processed within 24 h. The sampling procedure and the overall protocol used in this study is schematically represented in Figure 1.

### Sample preparation

#### Sample preparation for flow cytometric and plating studies

The bags were massaged vigorously by hand for 1 min and subsequently stroked in a lab blender (BagMixer® 400 P, Interscience, Saint Nom la Bretèche, France) at a fixed speed of 8 strokes/s for 30 s to release the cells into the PBS dispersion medium. The sponges were then forcefully squeezed by hand and the resultant suspension was transferred into a sterile beaker, representing three composite samples, one from each zone. Clarification of the suspension and separation of cells from debris were achieved by three-step centrifugation followed by a single-step filtration. The cell suspension from each composite sample was first sub-sampled into six polypropylene conical skirted centrifuge tubes (Sarstedt, Wexford, Ireland) and centrifuged at 300 × g for 5 min at 4°C using a Sorvall Legend RT centrifuge (Thermo Electron Corporation, Waltham, USA) to remove the large dust particles and debris. The supernatant was subsequently transferred to a clean centrifuge tube and centrifuged at 4000 × g for 20 min in order to harvest the cells. Finally, the supernatant was transferred again to a clean tube and centrifuged at 4000 × g for 45 min to harvest the possible remaining cells. For the first two steps of centrifugation, the acceleration and breaking were set at 5 and 7, respectively, while for the last step they were set at 9 and 1, respectively. The pellets from steps one, two and three of centrifugation were dispersed in 0.5, 5, and 0.5 mL of 4°C PBS, respectively. The dispersed pellets of the second, third and first stages of centrifugation were then filtered sequentially through sterile Whatman™ Grade 113V prepleated qualitative filter papers (GE Healthcare, Little Chalfont, UK) with pore size of ~30 μm. This was done to ensure that the final sample was free from large particles which could block the fluidics system of the flow cytometer. The filtrate (final sample volume of ~32 mL) was then transferred into a clean centrifuge tube and kept at 4°C before analysis within 24 h.

#### Sample preparation for 16S sequencing study

For 16S sequencing studies, the sample preparation was similar to that mentioned above with minor modifications. For each centrifugation, 50 ml of the suspension was used. Following the first centrifugation step (300 × g for 5 min at 4°C) and the removal of large dust particles and debris, the supernatant was transferred to a new 50 mL centrifuge tube and centrifuged at 10,000 × g for 10 min at 4°C to harvest the cells. For both steps of the centrifugation, the acceleration and breaking were set at 9. The supernatant was discarded and pellet was resuspended in 1 ml sterile PBS and transferred to a new 1.5 ml microcentrifuge tube. The two centrifuge steps were repeated until all the suspension in the sampling bag was centrifuged. The cell suspensions were stored at −80°C until analyzed.

### Flow cytometric study

#### Instrument

A BD FACSCanto II flow cytometer (BD Bioscience) equipped with green (488 nm air-cooled solid state; 20 mW laser output) and red (633 nm HeNe; 17 mW power output) lasers was used in this study. The fluorescence detector/filters relevant to this study were FITC (FL1; 530 ± 30 nm), PerCP-Cy5.5 (FL3; > 670 nm), APC (FL5; 660 ± 20 nm), and APC-Cy7 (FL6; 780 ± 60 nm). FSC and SSC detectors were also used for determining the light scatter parameters of forward scatter and side scatter, respectively. The instrument was cleaned before and after use and its performance was validated according to the manufacturer's instructions. In order to differentiate between the background noise and the signal (particles of interest), the threshold channel number was set at 200 on FSC. The number of background events (noise) detected by the instrument upon analysis of deionized water (deH_2_O) at a fast flow rate (≈ 80 μL/min) was less than 4 events/min (approximately less than 1 noise particle per 20 μL deH_2_O) (data not shown). For each parameter, the height, width and area (integral) of the voltage pulses of each event was measured and recorded.

#### Determination of the flow rate

The exact flow rate of the instrument was determined on the day of the experiment. Ten flow tubes (Sarstedt) were filled with 1 mL of deH_2_O. Each sample was acquired on the flow cytometer for 30 to 600 s at low, medium or high flow rate settings. The weights of the tubes were determined before and after analysis with the help of an analytical balance with accuracy of ± 0.001 g (Denver instruments, Göttingen, Germany). Assuming the density of deH_2_O to be 1000 kg/m^3^, a calibration curve was generated by plotting time vs. volume, to calculate the flow rate (μL/s) of the instrument. Therefore, by knowing the time required for recording a certain number of cells within a sample, it was possible to determine the volume, hence the flow rate of the instrument using Equation 1 (see Supplementary Figure 1).

(1) $$\begin{array}{l} Flow\;rate\;\left(\frac{\mu L}{min}\right)=\;\frac{\begin{array}{c} Number\;of\;beads\;counted\;\times \\ Sample\;volume\;\left(1,000\;\mu L\right) \end{array}}{\begin{array}{c} Acquisition\;time\;\left(1\;min\right)\times \\ number\;of\;beads\;added\;\left(5,110\;beads\right) \end{array}} \end{array}$$

#### Identification of the cells and gating strategy

**Figure 2:**
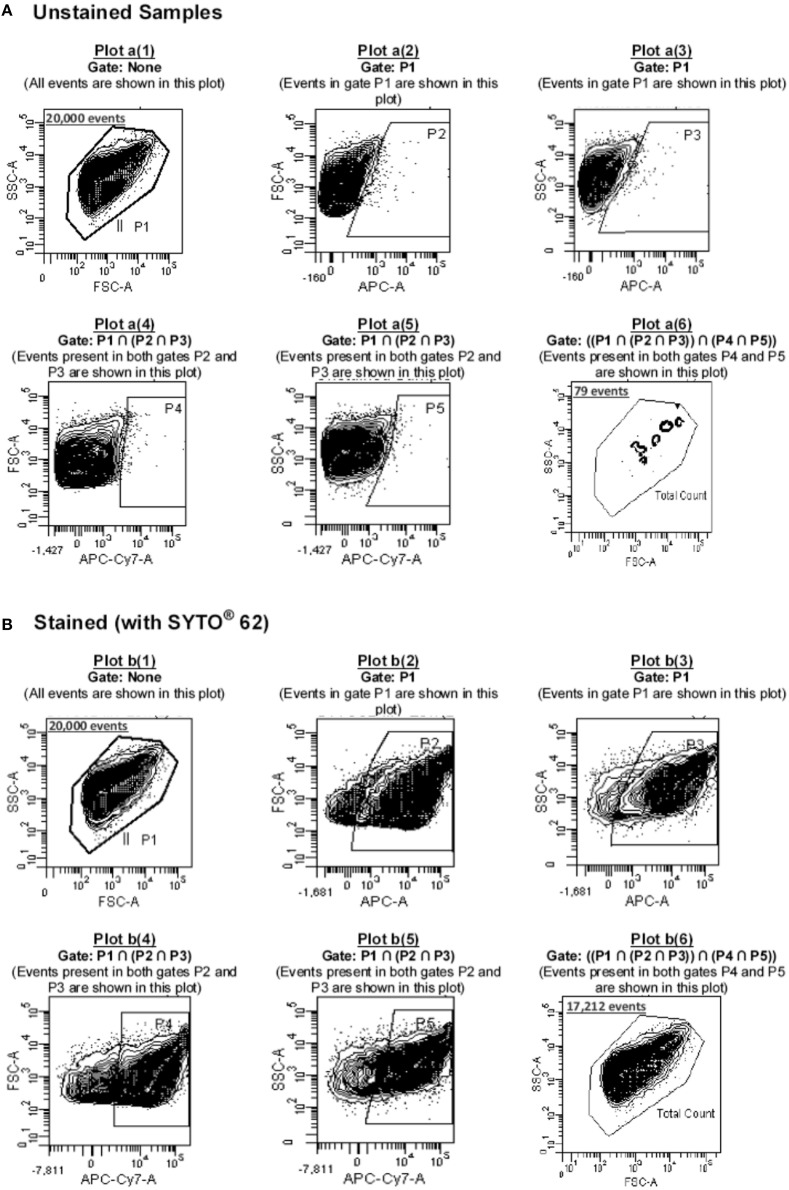
**Gating strategy used in this study**. Cells were acquired **(A)** before and **(B)** after staining with SYTO 62 dye. Based on Boolean logic, the events recorded within P1 (20,000 events) were passed through a series of gates (P2-P5) as shown in plots a(2)-a(5) (for unstained cells) and b(2)-b(5) (in the case of stained cells) to determine the number of noise particles (particles in gate P6 of plot a[6]; i.e., 79 noise particles) as well as cells of interest (particles in gate P6 of plot b[6] minus those shown in gate P6 of plot a[6], i.e., 17212–79 = 17133 cells).

Depending on the parameter and/or the type of fluorophore used in the study, the Photomultiplier tube (PMT) voltage for each parameter was adjusted so that the cells could be displayed in the center of the investigating plot. The samples were diluted to ensure that the flow rate was between 800 and 1200 events/s at medium speed setting (~45 μl/min). After displaying the data in a density plot of FSC vs. SSC and identifying the particles of interest, the latter was defined by creating a gating region (P1) around it, based on the light scatter properties of the particles (Plot a[1] and b[1]; Figure 2).

The defined gated population of P1 contained not only the presumed cells but also acellular particulates. Therefore, in order to differentiate between the two and detect the cells of interest, samples were stained with ~162 nM SYTO62 (Molecular Probes, Eugene, USA). This dye is a cell-permeant nucleic acid stain which is capable of staining most live and permeabilized (i.e., dead) bacteria. Its maximum excitation and emission wavelengths (λ_max_) are 652 and 676 nm, respectively. Considering the fact that SYTO62 was the only fluorophore used in this study that could primarily be excited by the red-laser, this made it possible to make exclusive use of the red-laser and its two detectors, FL5 and FL6 for detection of SYTO62-positive particles, hence the cells of interest. The principle behind the multi-gating strategy used in this study and using both FL5 and FL6 for detecting SYTO62 positive particles was based on the one reported by Buzatu et al. (2014). As the λ_max_ emission of SYTO62 is 676 nm, it is primarily detected by FL5; however, due to its broad emission spectrum (620–800 nm), its fluorescence could also be detected by FL6 detector (with relative fluorescence intensity of less than 24% compared to the λ_max_ emission wavelength). The gating strategy used in this study is described in details in Supplementary Data 2.

#### Determination of cell density and signal to noise (S/N) ratio

The cell density in the sample was calculated using the following equation:

(2) $$\begin{array}{r} Cell\;denisty\;\left(\frac{Cells}{cm^{2}}\right)=\frac{\left[\left(P6_{\left(Stained\right)}-P6_{\left(Unstained\right)}\right)\;\left(Cells\right)\right]\times Sample\;volume\;\left(mL\right)\times 1000\;\left(\frac{\mu L}{mL}\right)\times 60\;\left(\frac{s}{min}\right)}{Flow\;rate\;{\left(\frac{\mu L}{min}\right)}^{*}\times Acquisition\;time\;\left(s\right)\times Area\;sampled\;\left(cm^{2}\right)\times dilution\;factor} \end{array}$$

P6_(*stained*)_ and P6_(*unstained*)_ refer to the number of events within gate P6 for stained and unstained samples, respectively. By knowing the number of events for P6_(*unstained*)_ (i.e., noise particles), it was also possible to determine the S/N ratio by using the following equation:

(3) $$\begin{array}{l} Signal\;to\;noise\;ratio\;\left(\frac{S}{N}\right)=\\ \frac{Number\;of\;signal\;events\;\left(cells\right)\;within\;P6}{Number\;of\;noise\;events\;within\;P6} \end{array}$$

#### Physiological studies and staining strategy

Considering the heterogeneity of the microbiome in environmental samples and the variations between the stainability of different microorganisms with various dyes, it was decided to take a holistic multi-staining approach for studying the physiological status of the cells by staining the samples with a wide range of fluorescent dyes. Immediately prior to staining, 250 μL of diluted sample was transferred to 12 × 25 mm flow tubes and supplemented with 20 μL of filter-sterilized 100 mM EDTA (Sigma, Wicklow, Ireland) and 20 μL of 0.1% (v/v in deH_2_O) polyoxyethylene sorbitan monolaurate (Tween® 20) (Sigma) to improve the stainability of the cells.

The staining protocol for all the fluorochromes used in this study is shown in Table 1. In summary, samples were first stained with 10 μL of 5 μM working solution of SYTO62 to differentiate between the cells of interest and acellular particulates, as previously described. Samples were then vortexed for 1–2 s and incubated at room temperature (18 to 22°C) in darkness for 30 min before analysis. Upon identification of the cells of interest, SYTO62-stained sub-samples were also stained with the following combination of dyes to determine the physiological status of the cells: (a) Propidium iodide (PI) and SYTOX Green Dead Stain to study the membrane integrity; (b) PI in combination with SYTO9 or thiazole orange (TO) to determine the membrane integrity and viability; (c) PI with DiBAC_4_(3) (Bi-oxonol or BOX) to investigate the membrane potential and viability; (d) Fluorescein diacetate (FDA) and its derivatives [5-(and-6)-carboxyfluorescein diacetate (cFDA) and 5(6)-carboxyfluorescein diacetate N-succinimidyl ester (cFDA-SE)] as indicators of esterase activity; (e) 5-Cyano-2,3-di-(p-tolyl)tetrazolium chloride (CTC) for semi-quantitative analysis of the respiratory activity; and (f) Hexidium iodide (HI) in combination with SYTO9 for Gram staining. All samples were protected from light during the staining process. The protocols used for preparing the fluorophore stock and working solutions as well as the rationale behind using each one is described in detail in Supplementary Data 1.

**Table 1:** **The staining protocol used in this study**.

**Fluorophore**	**Concentration of the working solution**	**Volume (μL) of working solutions added to the sample**	Final concentration (M) of the dye in the sample^*^^†^	Incubation Time and temperature (post-staining, pre-analysis)^**^
SYTO^®^ 62	5 μM	10	166.67 nM(1)	30 min (RT)
			163.93 nM(2)	
			161.29 nM(3)	
			158.70 nM(4, 5, 6, 7)	
PI	299.23 μM	10	9.50 mM(4, 5, 6, 7)	5 min (RT)
BOX	19.36 μM	5	307.30 nM(5)	5 min (RT)
SYTO^®^ 9	250 μM	5	4.03 μM(3)	15 min (RT)
			3.97 μM(5)	
TO	42 μM	5	666.67 nM(6)	10 min (RT)
FDA	2.40 mM	5	39.37 μM(2)	30 min (37°C)
cFDA	2.50 mM	5	40.98 μM(2)	30 min (37°C)
cFDA-SE	25.11 nM	5	411.70 pM(2)	30 min (37°C)
SYTOX Dead	3 μM	5	49.18 nM(7)	20 min (RT)
HI	25.13 μM	5	405.32 nM(3)	15 min (RT)
CTC	53.46 mM	30	4.86 μM(8)	30 min (37°C)

*RT: Room temperature (18 to 22°C)*.

* *250 μL cell suspension, 20 μL of 100 mM EDTA, 20 μL of 0.1% Tween® 20*.

** *For instance, in the case of SYTO® 62/SYTO® 9/PI staining, cells were first stained with SYTO® 62 (at time 0 min), followed by staining with SYTO® 9 and PI at time 15 min and 25 min, respectively*.

† *The final concentration of the fluorochrome in the sample when stained with (1) SYTO® 62 only; (2) SYTO® 62/cFDA, SYTO® 62/cFDA or SYTO® 62/cFDA-SE; (3) SYTO® 62/SYTO® 9/HI; (4) SYTO® 62/PI/BOX; (5) SYTO® 62/PI/SYTO 9; (6) SYTO® 62/PI/TO; (7) SYTO® 62/PI/SYTO X®; and (8) SYTO® 62/CTC*.

#### Color compensation

Live and heat-killed samples of one Gram positive (*Lactobacillus rhamnosus* GG; LGG) and one Gram negative (*Escherichia coli;* EC) strain were used as compensation controls. The color compensation was performed for each fluorophore and its primary detector by first plotting a contour plot of the primary detector (the one used for measuring the fluorescence intensity of the fluorophore) vs. non-primary detector. The values for each fluorescence parameter on the plots were transformed bi-exponentially using the BD FACSDiva software version 6.1.3 (BD Biosciences). After gating the negative (non-fluorescent) and positive (fluorescent) cells, the median fluorescence intensity (FI) of the observed populations in primary and non-primary detectors was measured. When the FI of the positive cells in the non-primary detectors was larger than the one for negative cells, a percentage of the FI of the primary detector was subtracted from the affected non-primary FI in order to remove the spillover. The approximate values required for color compensation were calculated using the following equation:

(4) $$\begin{array}{r} Spillover\;correction\;\left(\%\right)=\frac{{\left(median\;FI\;of\;positive\;control-median\;FI\;of\;positive\;control\right)}_{non-primary\;detector}}{{\left(median\;FI\;of\;positive\;control-median\;FI\;of\;positive\;control\right)}_{primary\;detector}} \end{array}$$

The color compensation was performed using the calculated values and verified by visualizing the effects of the applied values on the median FI of the non-primary detectors. If necessary, using the calculated value as a guide, an arbitrary percentage of the FI of the primary detector was subtracted from the affected non-primary FI until the median FI of the latter was relatively the same for both the positive and negative controls. The information on the control samples and the color compensation values used in this study could are shown in Supplementary Tables 2, 3, respectively.

#### Analysis and display of FCM data

The data were acquired and analyzed using BD FACSDiva software version 6.1.3. For FSC vs. SSC contour plots, the scale of the axis for each parameters was logarithmic. When samples were dual-stained (including SYTO62), histograms were used for displaying the FL1 (in the case of FDA, cFDA, cFDA-SE) or FL3 (for CTC). For histograms, the scales for the x-axis (fluorescence channel number) were transformed bi-exponentially (logical x-axis) while the y-axis (count) was displayed on a linear scale beginning at zero. In the case of triple-staining (including SYTO62), the channel numbers (x-axes) for all the fluorescence parameters were transformed bi-exponentially and plotted in two-dimensional quantile (probability) contour plots. All contour plots displayed the events with 99% probability as well as the outliers. The gates (rectangles, polygons, quadrants and vertical or horizontal markers) for each sub-population were set manually.

### Plating study

#### Growth media

The growth media used in this study consisted of M9 minimal salt media agar (M9), nutrient agar (NA) (Oxoid, Altrincham, UK) and Brain Heart Infusion (BHI) agar (Sigma). M9, NA, and BHI were used as low, medium and rich nutrient media, respectively, and were used to investigate the effects of the nutrient content of the growth medium on recovery of cells from the processing environment samples. Unless stated otherwise, all media and diluents were prepared according to the manufacturer's instructions using distilled water and autoclaved at 121°C for 15 min. After autoclaving, the salt and agar solutions were allowed to cool to 50°C. The salt broth was then supplemented with 20 mL of 20% (w/v) D-(+)-glucose (Fisher Scientific, Loughborough, UK), 2 mL of 1 M MgSO_4_ (Fisher Scientific), 0.2 mL of 0.5 M CaCl_2_ (Reagecon, Shannon, Ireland), and 0.1 mL of 0.5% (w/v) thiamine hydrochloride (Sigma-Aldrich). All the supplements for M9 agar were filter sterilized using 0.22 μm syringe filters (Sarstedt). M9 minimal agar was finally prepared by mixing 500 mL of the supplemented M9 salt broth with 500 mL of 3% (w/v) sterile agar solution.

#### Recovery of viable but non-culturable cells (VBNC)

Prior to pouring as agar plates, M9, NA and BHI were also occasionally supplemented with the reactive oxygen species (ROS) scavengers, catalase (Sigma), and sodium pyruvate (SP) (Sigma-Aldrich). To investigate the possible resuscitation of injured and stressed VBNC cells, the supplementation of the agar plates (≈ 20 mL) with 2000 units of catalase per plate (100 μL stock solution) or 0.3% of SP (200 μL stock solution) was performed around 10 min prior to plating the sample. Respectively, 20,000 units/mL and 300 mg/mL stock solutions of catalase and SP were prepared by vigorous vortexing of the compounds in distilled water followed by filter sterilization with 0.22 μm filters. Stock solutions were prepared on the day of experiment and stored at 4°C until use.

#### Total aerobic count (TAC)

In order to determine the total aerobic count (TAC), samples were first decimally serially diluted in maximum recovery diluent (MRD; Fluka), allowed to stand for 30 min after which 100 μL of each dilution was plated on the aforementioned growth media. The plates were incubated either at the room temperature (≈ 21°C), 30°C or 37°C for 48 h. The colonies were examined and counted after 24 h and 48 h incubation.

### 16S rDNA sequencing study

#### Genomic DNA extraction and quality check

In this study, the processing environment samples were collected from a PIF production site which had strict sanitary standards; therefore, they contained far less cells compared with environment samples collected from the natural environment such as water and soil. As a result, commercial kits with filter column genomic DNA extraction was not used, to avoid filter column clogging and genomic DNA loss during the binding-washing step. Genomic DNA was extracted using a chloroform-based method. Samples (see Section Sample Preparation for 16S Sequencing Study) were centrifuged at 8000 × g, at room temperature for 2 min, the supernatant was discarded and the pellet was resuspended in 1 ml sterile PBS. This washing step was repeated three times after which the pellet was resuspend in 300 μl DNase/RNase free H_2_O. The resuspended pellet was boiled at 100°C on a heating block for 5 min. After boiling, it was vortexed for ~5 s to ensure the disruption of cell walls and then centrifuged at 8000 × g, for 5 min at room temperature. Subsequently, the supernatant was transferred to a new microcentrifuge tube, mixed with chloroform (Sigma) in a 1:1 ratio and vortexed for 5 s to ensure thorough mixing of the aqueous and chloroform phases. Finally, the mixture was centrifuged at 13000 × g, 4°C for 10 min and ~75% of the aqueous (upper) phase that contained genomic DNA was transferred to a new tube. Nanodrop® Spectrophotomer ND-1000 (1 μl genomic DNA) and Qubit® 2.0 Fluorometer (1 μl genomic DNA; Thermo Fisher Scientific) were used to check genomic DNA concentration and quality, and the genomic DNA was stored at −20°C for further use.

#### Total RNA extraction

As with DNA extraction, no commercial kit was used to avoid filter column clogging and total RNA loss during the binding-washing step. Samples were centrifuged at 8000 × g, at room temperature for 2 min, the supernatant was discarded and the pellet was resuspended and washed with 1 ml sterile PBS. The centrifuge and wash steps were repeated twice and the pellet was resuspend in 1 ml sterile PBS, transferred to a 50 ml centrifuge tube and sterile PBS was used to adjust the final volume of the cell suspension to 4 ml. To this suspension, 1.6 mL of ice cold phenol-ethanol solution (95% ethanol and 5% acidic phenol; pH 4.3) was added and the tube was incubated on ice for at 30–120 min to stabilize the RNA and prevent degradation (Tedin and Bläsi, 1996). The mixture was centrifuged at 3300 × g at 4°C for 10 min and most of the supernatant was discarded. The pellet was resuspended with the remaining supernatant in the tube and transferred to a 1.5 ml tube. The tube was centrifuged at 18,000 × g, 4°C for 1 min, the supernatant was discarded and the pellet was resuspended in 1 ml of TRIzol (Ambion, Foster City, USA). The mixture was transferred to a 2 ml heavy phase lock tube (5 Prime) and supplemented with 400 μl chloroform (Sigma-Aldrich). The tube was immediately gently inverted for 10 s (no vortexing) and incubated at room temperature for 2–5 min. The mixture was centrifuged at 16,000 × g, at room temperature for 15 min and the aqueous phase was transferred to a new microcentrifuge tube, supplemented with 450 μl isopropanol (Sigma-Aldrich) and mixed immediately. The mixture was incubated at room temperature for 20 min and stored at −20°C overnight to provide higher yields. After storage, the mixture was centrifuged at 16,000 × g at room temperature for 30 min, the supernatant was discarded, the pellet was washed with 350 μl of 70–75% ethanol and centrifuged at 16,000 × g at room temperature for 10 min. After air drying the pellet, 25 μl of pre-heated (65°C) DNA/RNase-free H_2_O was added and the tube was incubated on a thermomixer at 900 rpm, 65°C for 5 min. During this time, the tubes were vortexed briefly 2–3 times to improve the dispersion of the pellet. The liquid in the tube contained total RNA. DNA removal was carried out using Ambion TURBO DNA-free™ kit (Thermo Fisher Scientific, USA). An Agilent 2100 Bioanalyzer (1 μl total RNA) and a Nanodrop® Spectrophotomer ND-1000 (1 μl total RNA) were used to check total RNA quantity and quality, and the sample was stored at −80°C for further use.

#### 16S rDNA sequencing and bioinformatics analysis

16S rDNA sequencing was carried out using the Illumina MiSeq platform. Such sequencing is amplicon-based, targeting the variable region V3-V4 of the 16S rDNA gene and generates PCR products with a length of ~460 bp. Paired-end, 300 × 2 bp sequencing was carried out to cover the whole PCR product from the two opposite ends. For library construction, the Illumina official guide for 16S rDNA sequencing library preparation was used as reference. Bioinformatics analysis was carried out using the 16S Metagenomics app v1.0.1 provided in the Illumina BaseSpace online platform, including reads quality control, reads alignment, assembly and annotations. For taxonomic classification of the 16S rDNA reads, the Ribosomal Database Project (RDP) Classifier was used for classification (Wang et al., 2007) and an Illumina-curated version of the GreenGenes taxonomic database was used as reference database (DeSantis et al., 2006). Both applications were inbuilt in BaseSpace.

## Results and discussion

### Flow cytometric and plating studies

Table 2 shows the FCM data obtained for three samples collected from low, medium and high care zones. The greatest number of cells (regardless of their physiological state) was detected in the low care zone, followed by the medium and high care zones (*p* < 0.0001). This was expected considering the implementation of stricter levels of hygiene and working practices by the PIF manufacturer in the latter two zones. However, the reduction in the total cell count, did not necessarily translate to a concurrent reduction in the number of viable cells as determined by FCM. By knowing the total cell count and the percentage of viable cells (based on the exclusion of PI viability dye), it was possible to calculate the density of viable cells per cm^2^ of the sampling zone. The number of viable cells per cm^2^ in medium care was nearly 3 times greater than that detected in both low and high care zones. This was probably due to greater level of humidity, hence greater access of microorganisms to available water in this zone. The lack of significance between the FCM viability results obtained for the two dry zones (low and high care) could be considered as further evidence of the primary role of humidity in improved viability of the cells in the wet medium care zone.

**Table 2:** **Flow cytometric (FCM) total cell and viable cell density**.

	**Low care**	**Medium care**	**High care**
**KNOWN VALUES**			
(A) Sample volume (mL)	32	33	33
(B) Dilution factor (ratio of sample to PBS)	1:12	1:12	1:6.6
(C) Area sampled (cm^2^, approximately)	50000	50000	50000
**ACQUIRED FCM DATA**			
(D) Number of data recorded in gate P1	20000	20000	20000
(E) Number of SYTO® 62-stained particles in gate P6	16931 ± 398	13548 ± 33	6857 ± 194
(F) Number of unstained particles in gate P6	79 ± 0	71 ± 3	47 ± 3
(G) Number of presumed cells in P6 (E−F)	16852 ± 398	13477 ± 33	8610 ± 194
(H) Percentage noise particles (F/G)	0.47 ± 0.01%	0.53 ± 0.02%	0.55 ± 0.04%
(I) Percentage of presumed cells in P1 (G/A)	84.26 ± 1.99%	67.38 ± 0.17%	43.05 ± 0.97%
(J) Acquisition time (s)	186 ± 19	226 ± 11	193 ± 59
(K) Flow rate (μL/min)	45.82	45.82	45.82
**TOTAL CELL DENSITY [L**_1_ = **(G** × **A** × **1000** × **60)/(K** × **J** × **C** × **B)]**			
(L_1_) cells/cm^2^	917 ± 73^*X*^	621 ± 31^*Y*^	267 ± 77^*Z*^
(L_2_) log_10_ cells/cm^2^(^*^)	**(2.96 ± 0.03)**	**(2.79 ± 0.02)**	**(2.43 ± 0.12)**
(M) Percentage viable (*n* = 8)^(^**^;*See* Table 3*for calculations*)^	4.53 ± 2.71%	19.09 ± 3.12	16.12 ± 5.36
**VIABLE CELL DENSITY (L**_1_ × **M)**			
(N_1_) cells/cm^2^	42 ± 25^*X*^	118 ± 20^*Y*^	43 ± 19^*X*^
(N_2_) log_10_ cells/cm^2^(^*^)	**(1.62 ± 0.26)**	**(2.07 ± 0.07)**	**(1.63 ± 0.19)**

*Unless stated otherwise, the values shown here are the mean ± standard deviation (SD) of two technical replicates*.

* : The SD values are the differential relative errors, i.e. absolute standard deviation divided by the mean value and multiplied by 0.434; e.g., 0.434 × (79/917) = 0.03;

** *: Viable cells were considered as those with intact membrane which either excluded PI (when used in combination with either of SYTO 9, TO or BOX) or were not stained with SYTOX Green Dead Stain dye (n = 8, two technical replicates per dye combination: SYTO 9/PI, TO/PI, BOX/PI and SYTOX Green only). In each row, the values with similar letters (X–Z) are not statistically significant (both p > 0.05; based on unpaired two-tailed Student's t-test) (Data from sampling in October 2015)*.

**Figure 3:**
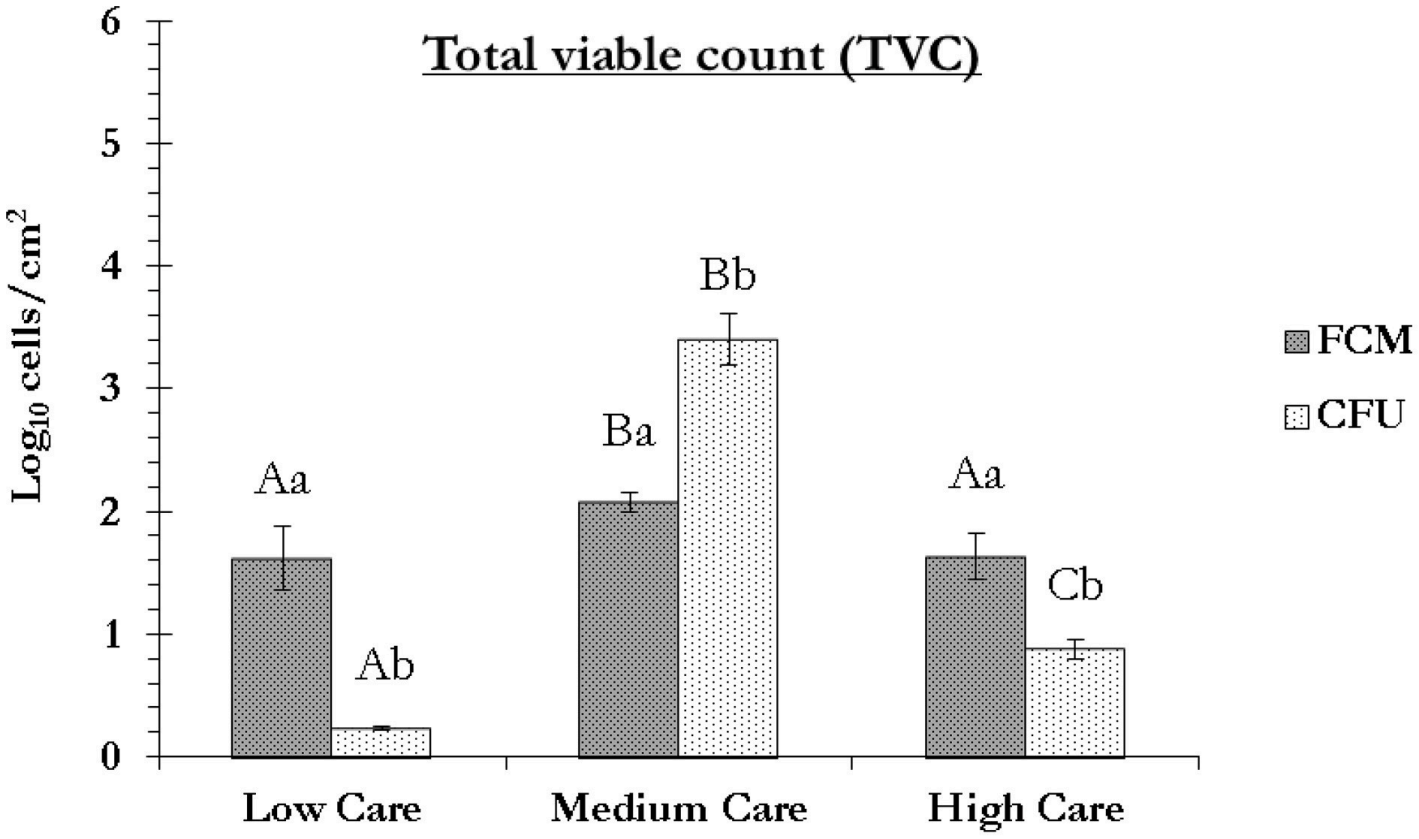
**Total viable count (log_**10**_ cells/cm^**2**^) based on the flow cytometry (FCM) and plate counting (CFU) techniques**. For FCM, a cell was considered as viable if it could not be stained with either of PI or SYTOX Green Dead Stain, while for CFU, it formed a colony on nutrient agar plate at 37°C, 48 h post-inoculation. The FCM values are the same as those calculated in row N of Table 2. All the values are the mean ± SD of two technical replicates. Capital letters are used for comparing the FCM or CFU data between each zone, while lower-case letters compare the FCM and CFU values within each zone. Columns marked with similar capital or lower-case letters are not statistically significantly different based on unpaired two-tailed Student's *t*-test; *p* > *0.05*) (Data from sampling in October 2015).

The results of the flow cytometry study were in agreement with those of the plate counting technique in the sense that the cells from medium care exhibited significantly greater culturability as well as viability compared to those from the other two care zones (Figure 3). In both dry zones, compared to the FCM, the plate counting significantly under-estimated the number of viable cells, while in the medium care, significantly lower number of cells were detected by FCM, compared to the plate counting. The under-estimation of viable cells by plate counting due to the presence of stressed and starved VBNC cells is well-documented (Oliver, 2005) and was, therefore, expected. On the other hand, the under-estimation of viable cells by FCM in medium care may be due to the presence of ultramicrobacteria and ultramicrocells (e.g., bacteria of the genera *Flavobacterium, Bacteroides*, and *Chryseobacterium*) with sizes smaller than the detection limit of the flow cytometer used in this study (< 0.5 μm). This could have resulted in the cells being considered as background noise. This could also mean that the number of FCM viable cells, hence VBNC cells in dry care zones of low and high could have been significantly higher than determined in this study. Moreover, the possible presence of very high concentration of surfactants, detergents and washing solutions in that zone, could have rendered some of the cells un-stainable with SYTO62, hence not detectable based on the proposed protocol (Vives-Rego et al., 1999).

Table 3 shows the FCM data regarding the physiological status as well as Gram characteristics of the cells in each care zone. With regard to membrane integrity, membrane polarization, and metabolic activity, relatively similar results were obtained. The greatest mean percentage of cells with intact and polarized membranes and metabolic activity was observed in medium care, followed by high and low care zones. For instance, with the exception of SYTO9/PI staining, the percentage of cells with intact membranes was significantly lower in low care compared to the other two zones, regardless of the fluorophores used. The discrepancies observed between the results obtained for each fluorophore combination could be due to the difference between their staining mechanism (Netuschil et al., 2014). Although, the greatest percentage of cells with respiratory activity was found in the medium care sample, the values obtained were significantly higher than the percentage of viable cells. The reason behind this observation is not clear, however, it could be due to the residual activity of the electron transfer chain in cells with depolarized and compromised cells. Moreover, the difference between the stainability of Gram negative and Gram positive cells could have played a role in the discrepancies observed (Holm and Jespersen, 2003).

**Table 3:** **The physiological status and Gram characteristics of the microbiome**.

**Physiological status**	**Low care**		**Medium care**		**High care**	
(a) Intact membrane (*n* = 8; I-IV)^*^	4.53 ± 2.71%	X	19.09 ± 3.12%	Y	16.12 ± 5.36%	Y
i.SYTO9^+^/PI^−^ (*n* = 2)	1.72 ± 0.04%	A;X	18.83 ± 1.58%	AB;Y	9.33 ± 0.59%	A;Z
ii.TO^+^/PI^−^ (*n* = 2)	2.70 ± 0.03%	B;X	16.72 ± 0.19%	B;Y	20.52 ± 1.76%	B;Y
iii.BOX^±^/PI^−^ (*n* = 2)	8.17 ± 0.48%	C;X	23.76 ± 1.59%	AC;Y	20.75 ± 3.57%	BCD;Y
iv.SYTOX^−^ (*n* = 2)	5.53 ± 0.42%	D;X	17.04 ± 0.02%	B;Y	13.87 ± 1.05%	C;Y
(b) Polarized membrane (*n* = 2; BOX^−^)	0.27 ± 0.06%	X	10.29 ± 0.04%	Y	1.46 ± 0.02%	Z
(c) Metabolically active (*n* = 4; VI-VII)^**^	3.50 ± 3.35%	X	20.08 ± 9.34%	Y	10.59 ± 2.29%	Y
v.FDA^+^ (*n* = 2)	0.25 ± 0.02%	E;X	2.80 ± 0.38%	E;Y	0.34 ± 0.01%	E;Z
vi.cFDA^+^ (*n* = 2)	6.04 ± 0.02%	F;X	28.01 ± 0.38%	F;Y	12.56 ± 0.15%	F;Z
vii.cFDA-SE^+^ (*n* = 2)	0.96 ± 0.03%	G;X	12.16 ± 0.44%	G;Y	8.62 ± 0.43%	G;Z
(d) Respiratory activity (*n* = 2; CTC^+^)	10.18 ± 0.46%	X	44.21 ± 3.56%	Y	1.38 ± 0.33%	Z
(e) Gram positive (*n* = 2; HI^+^)	12.40 ± 0.01%	X	19.70 ± 1.58%	Y	6.10 ± 2.35%	X

In each column, the values with similar letters (A–D for membrane integrity and E–G for metabolic activity data) are not statistically significant. Moreover, in each row, the values with similar letters (X–Z) are not statistically significant (both p > 0.05; based on unpaired two-tailed Student's t-test). With the exception of rows marked with ^*^ and ^**^, all the values are the mean ± standard deviation (SD) of two technical replicates.

* *: The values in row (a) are the mean ± SD of the percentage of cells that were not stained with either of PI or SYTOX Green (intact membrane; rows I to IV)*.

** *: The values in row (c) are the mean ± SD of the percentage of cells that were stained with either of cFDA or cFDA-SE (esterase activity; rows VI and VII respectively). The data obtained for FDA^+^ were not regarded for the purpose of this calculation, due to apparent passive leakage of the fluorescein dye from the cells (Data from sampling in October 2015)*.

**Figure 4:**
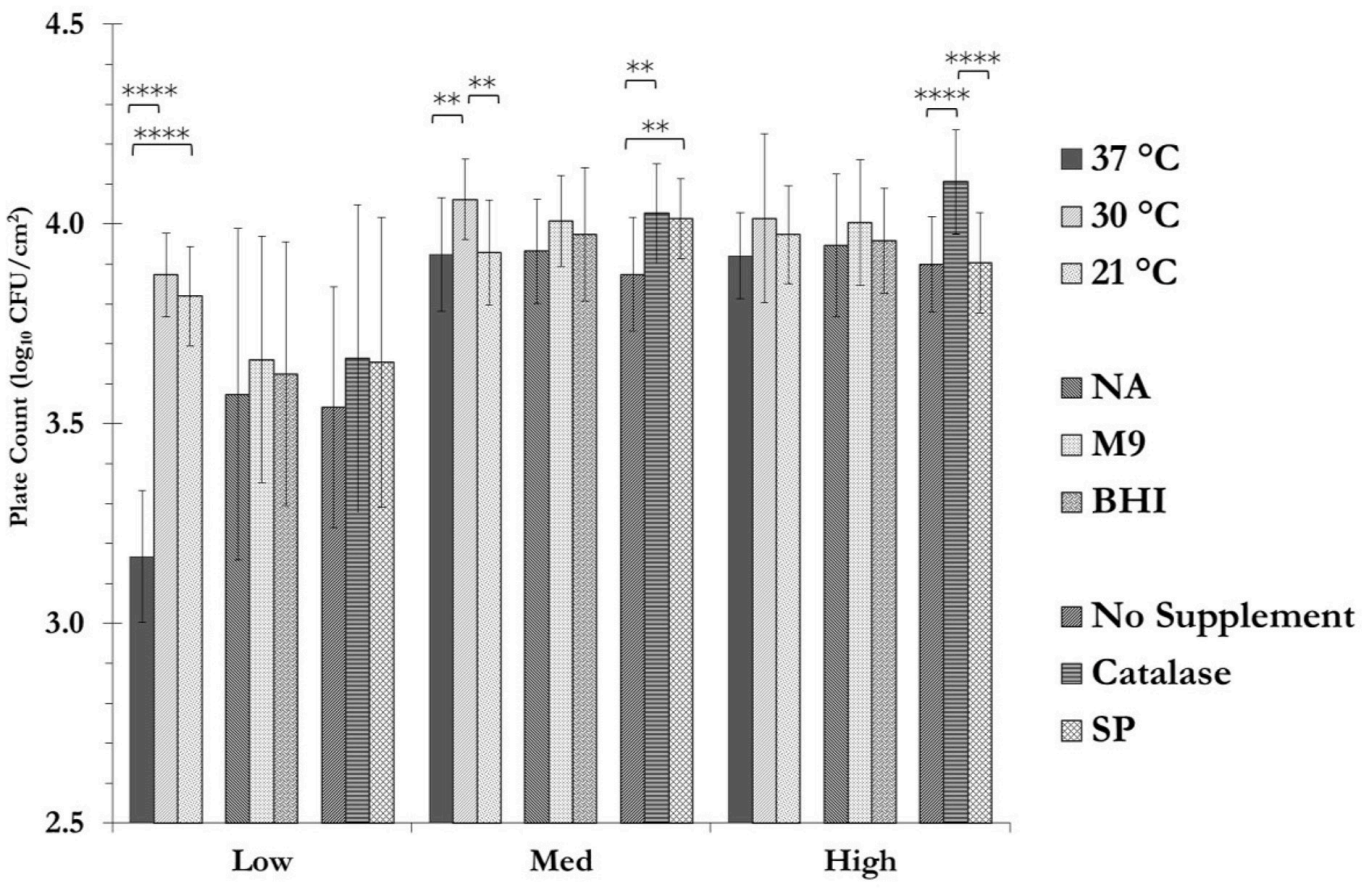
**Recovery of stressed cells using catalase and sodium pyruvate**. Samples were spread plated in duplicate on nutrient agar (NA), M9 Salt agar or Brain Heart Infusion agar (BHI) solid growth media (with or without catalase or SP) and incubated at either of 21, 30, or 37°C. For each temperature/sample combination (e.g., low care sample at 37°C) shown, the data are the mean ± SD (technical duplicate plating of a single sample) of the plate count obtained from all the plates that were subjected to the indicated parameter, regardless of the effects of others (*n* = 18). For instance, the plate count for the low care sample at 37°C (3.17 ± 0.17) is the mean ± SD of the plate counts for all the eighteen plates that were incubated at 37°C, regardless of the growth medium or supplementation. See Supplementary Table 4 for further information on the three-way interaction between temperature, media and supplemnetation variables for each sample (Data from sampling in May 2015). (^**^: *p* < 0.01; ^****^: *p* < 0.0001).

Changing the incubation temperature and or the supplementation of the growth media had a significant effect on the recovery of the cells on a care-zone basis (Figure 4 and Supplementary Table 4). For instance, decreasing the incubation temperature from 37 to 30°C or room temperature, had a highly significant effect on the total aerobic count for samples collected from the low care zone (*p* < 0.0001). This could indicate the presence of a significant number of psychrotrophic bacteria in low care samples (Hantsis-Zacharov and Halpern, 2007). The change in nutrient content of the media did not make a statistically significant difference in the recovery of the stressed cells. On the other hand, supplementation of the growth media with reactive oxygen species scavengers seemed to improve the recovery of the cells. Catalase and SP have previously been shown to be effective in recovery and resuscitation of injured and VBNC cells (Mizunoe et al., 2000; Bang et al., 2007). Catalase was effective in recovery of the cells collected from both medium and high care zones, while SP only improved the recovery in medium care samples. In the case of low care samples, the results were similar to those observed in the medium care zone, however, they did not reach the level of statistical significance.

### 16S rDNA sequencing study

The mean DNA content in low, medium and high care zones was 278, 168, and 53 pg/cm^2^, respectively. Considering the vast difference between the DNA content of different bacterial species, it was not possible to establish a direct correlation between the DNA content of the sample and the cell count. Nonetheless, the greatest DNA content was found in the low care sample, followed by medium and high care zone samples, which closely resembled the results for cell counts obtained using from the FCM total count, as previously described. In addition, the mean total RNA content for samples of low, medium and high care zones was 2, 29, and 1 pg/cm^2^, respectively. By making the presumption that the presence RNA in the cell is an indicator of protein synthesis, hence a degree of cellular vitality, the RNA content of the cells was in agreement with both the FCM viable count and plate counts.

**Figure 5:**
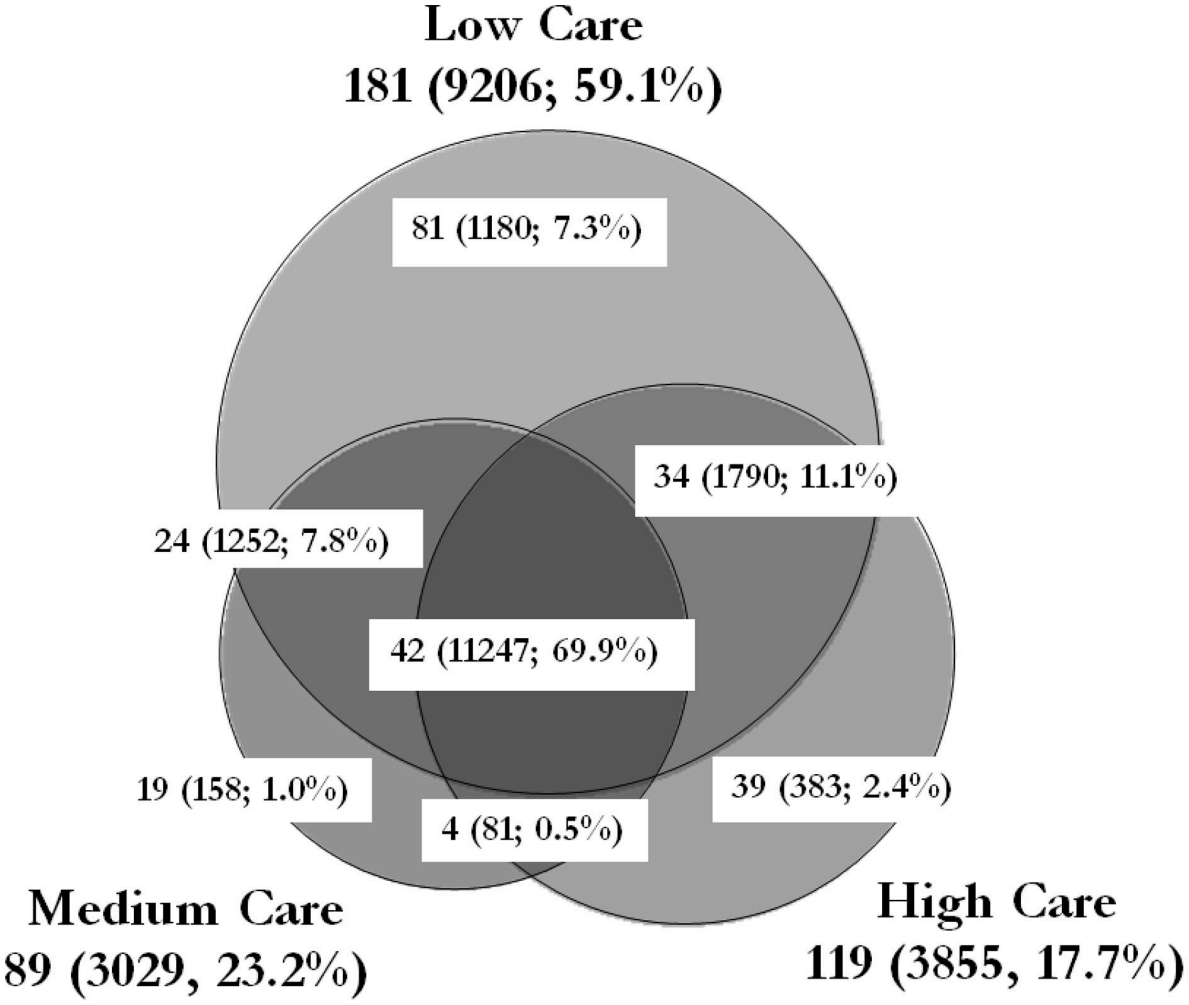
**Schematic representation of the number of genera identified in each care zone**. The size of each circle and the overlap areas are propotionate to the number of genera idnetified in that zone. The values outside the parantheses show the number of genera idenfied within the zone/area. The values within the parentheses show the total cells/cm^2^ and the percentage contribution of those genera to the overall microbiome of the PIF production unit. The total cell counts exclude the unpecified genera and/or species (2682, 540, and 819 cells/cm^2^ of unspecified bacteria in low, medium and high care zones, respectively). This Venn diagram was generated using the BioVenn software by Hulsen et al. (2008).

16S sequencing provided valuable information on the type of bacteria present in each care zone and the percentage contribution of each genera to the entire microbiome. This technique has been used successfully to characterize the microbiome in various environments such as soil, water, food and hospitals (Gomez-Alvarez et al., 2012; Oberauner et al., 2013; Rampelotto et al., 2013; de Boer et al., 2015). By integrating these data with that of the total FCM cell count, it was possible to calculate the number of cells belonging to each genera in each care zone. 16S sequencing of the samples revealed the presence of 243 bacterial genera (with more than 0.05% distribution) in the microbiome of the PIF production facility (Figure 5). The combination of 16S rRNA-targeted oligonucleotide probes with flow cytometry for analyzing mixed microbial populations has been reported (Amann et al., 1990), although there are no reports of its use to characterize the microbial population of a food production environment. The results pointed to a striking similarity between the type of bacteria present in different care zones. For instance, 42 out of 243 genera were common to all three care zone, contributing to nearly 70% of the microbiome. Similarly, 58 genera were common between low care and the other two care zone. On the other hand, although a third of the identified genera were unique to low care, they only made up 7.3% of the microbiome (total 1180 cells/cm^2^). Similarly, 19 and 39 genera were unique to medium and high care zones, making up 1.0 and 2.4% of the microbiome, respectively.

Looking at the top thirty genera, in terms of % occurrence, in the PIF production unit provided a better picture of the type of microorganisms associated with each care zone (Table 4). For the complete list, readers are referred to Supplementary Table 5. Twenty out of 30 genera which were predominantly present in low care zone, are mainly associated with soil and the general environment, which included species belonging to *Pseudomonas, Spirosoma*, and *Sphingomonas* genera. On the other hand, those predominantly present in wet care zone such as *Acinetobacter, Chryseobacterium*, and *Paucibacter* are mainly associated with water and sewage, as well as soil and other general environment sources. In contrast, the greatest number of human and milk-associated genera such as *Streptococcus, Lactococcus, Corynebacterium, Lactobacillus*, and *Kocuria* were found in the high care zone. Washing the sponges of the low care zone in PBS resulted in the formation of very dark gray cell suspension with a significant soil and debris sedimentation. Unlike the other two care zones, the majority of drains and sampling points (mainly drains) within medium care were wet. Based on the current data, it was not possible to definitively determine the primary reason behind the greater prevalence of human-associated microorganisms in high care zone. However, it is plausible that, while strict segregation of the high care zone led to a substantial reduction in the entry of soil and drain-associated microorganisms from the other two care zones, human-associated microorganisms within that zone still contributed to the microbiome of high care zone.

**Table 4:** **The top 30 genera in the PIF production unit (all care zones)**.

		**Low care**		**Medium care**		**High care**		
Overall ranking^*^	**Genus**	**Distribution (%)**	**Cell density (Cells/cm^2^)**	**Distribution (%)**	**Cell density (Cells/cm^2^)**	**Distribution (%)**	**Cell density (Cells/cm^2^)**	**Mean Density (Cells/cm^2^)**
1	Acinetobacter	5.64	670	**27.76**	**991**	2.90	136	599
2	Streptococcus	4.81	572	0.68	24	**24.88**	**1163**	586
3	Pseudomonas	**6.60**	**785**	13.02	464	4.82	225	492
4	Spirosoma	**5.39**	**641**	0.08	3	1.11	52	232
5	Sphingomonas	**4.69**	**558**	0.16	6	1.76	82	215
6	Lactococcus	1.47	175	2.03	72	**6.96**	**326**	191
7	Pedobacter	**3.39**	**403**	0.18	6	–	–	136
8	Chryseobacterium	1.29	154	**6.68**	**238**	0.20	9	134
9	Calothrix	**2.38**	**283**	–	–	2.01	94	126
10	Flavobacterium	**2.56**	**304**	0.49	17	0.71	33	118
11	Janthinobacterium	**2.20**	**261**	1.83	65	0.18	9	112
12	Enterobacter	0.17	21	**7.02**	**251**	0.98	46	106
13	Psychrobacter	0.90	107	**4.29**	**153**	0.81	38	100
14	Corynebacterium	0.75	89	0.16	6	**4.26**	**199**	98
15	Hymenobacter	**1.83**	**218**	–	–	0.24	11	76
16	Lactobacillus	0.70	83	0.16	6	**2.82**	**132**	74
17	Bacteroides	**1.25**	**148**	0.15	5	1.18	55	70
18	Paucibacter	0.36	43	**3.19**	**114**	0.20	9	55
19	Staphylococcus	**0.77**	**91**	–	–	1.49	70	54
20	Oxalobacter	**1.17**	**139**	0.19	7	0.25	12	53
21	Roseomonas	**1.22**	**145**	0.05	2	0.16	8	51
22	Arthrobacter	**0.73**	**87**	0.92	33	–	–	40
23	Sejongia	**0.83**	**99**	0.08	3	0.37	17	40
24	Kocuria	0.38	45	0.26	9	**1.25**	**58**	38
25	Stenotrophomonas	**0.67**	**79**	0.18	7	0.45	21	36
26	Bacillus	**0.60**	**71**	–	–	0.66	31	34
27	Dyadobacter	**0.71**	**84**	0.37	13	0.06	3	33
28	Novosphingobium	**0.77**	**92**	0.12	4	–	–	32
29	Tolumonas	**0.32**	**39**	1.02	36	0.29	13	29
30	Variovorax	**0.71**	**84**	0.08	3	–	–	29

* *: The ranking (out of 243 genera identified in all three zones, with euqal to or more than 0.05% distribution) is based on the calculated mean density of the specified genus in all three zones (i.e., the sum of the denisties in each zone divided by three as shown in the last column). In each row (genus), the highlighted in bold cell indicate the zone at which the greatest density for that genus was observed. See Supplementary Table 5 for the list of all genera identified and their distribution in each care zone (Data from sampling in May 2015)*.

**Figure 6:**
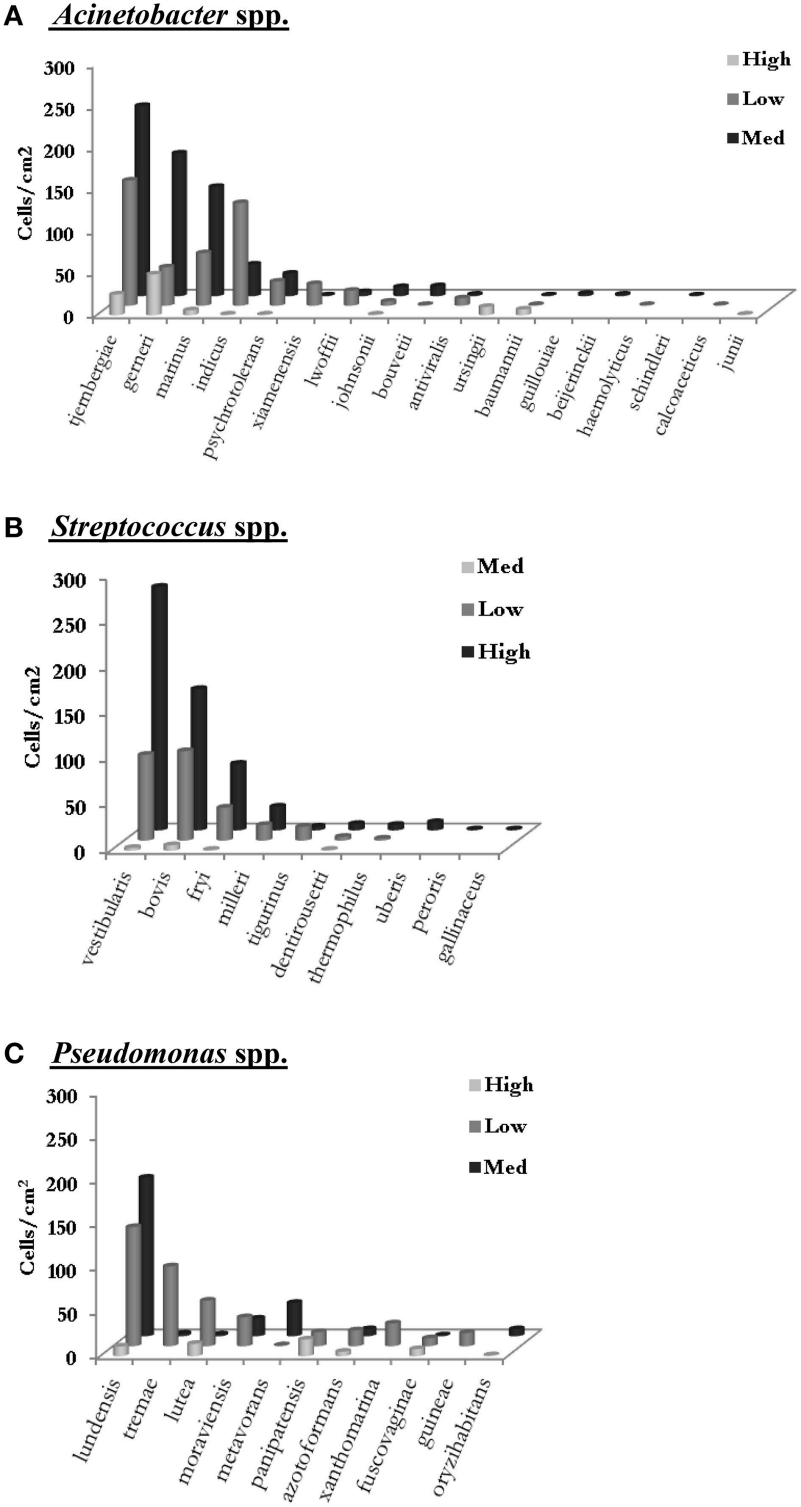
**The number of cells for each species of (A) ***Acinetobacter***, (B) ***Streptococcus*** and (C) ***Pseudomonas*** was determined by multiplying the percentage distribution of each species in each care zone (as determined by 16S rDNA sequencing) by the total cell count for the corresponding care zone (as determined by FCM) (Data from sampling in May 2015)**.

A closer look at the number of cells of the different species of the top three genera could be used as a good indicator of the possible transition of the cells between different zones (Figure 6). For instance, with regard to the *Acinetobacter*, the top five species of this genus in low care and the top three in low care were also among the top five species of this genus in medium care. Similar results were also obtained for *Streptococcus* spp. where *S. vestibularis, S. bovis*, and *S. fryi* were the top three species of this genus in all three zones. Furthermore, the rate of change in the number of cells for each species in one zone, closely resembled the change observed in the other two zones. It is important to note that the aim of this study was to compare the microbiome of different care zones and therefore, sponges from different locations of a specific care zone were placed in a single bag. Consequently, this could have contributed to the variability observed between the results for each care zone. Further studies are needed to determine the microbiome of each sampling point.

16S sequencing also provided information on the pathogenic strains present in each care zone. Table 5 shows the percentage distribution of 18 pathogenic species and 1 pathogenic genus in three different care zones. According to the official report of FAO/WHO (2004), these species are divided into three categories “based on the strength of evidence of a causal association between their presence in PIF and illness in infants.” Class A includes *Cronobacter* spp. and *Salmonella enterica* for which clear evidence of causality exist. No Class A microorganisms were detected in either of the three care zones. On the other hand, Class B (i.e., causality plausible, but not yet demonstrated) and Class C organisms (i.e., causality less plausible, or not yet demonstrated) were detected at both genus and species level in all three zones.

**Table 5:** **Pathogenic species identified in different care zones**.

	**Genus**	**Species**	**Low**	**Medium**	**High**
CLASS A	*Cronobacter*	*sakazakii*	ND	ND	ND
	*Salmonella*	*enterica*	ND	ND	ND
CLASS B	*Citrobacter spp*.		0.001%	**0.116%**	0.048%
	*Citrobacter*	*freundii*	ND	**0.057%**	0.014%
	*Citrobacter*	*koseri*	ND	ND	ND
	*Enterobacter spp*.		**0.173%**	**7.022%**	**0.978%**
	*Enterobacter*	*cloacae*	ND	0.001%	ND
	*Hafnia*	*alvei*	ND	ND	ND
	*Klebsiella spp*.		0.002%	**0.291%**	0.001%
	*Klebsiella*	*pneumoniae*	ND	0.001%	ND
	*Klebsiella*	*oxytoca*	ND	0.004%	ND
	*Pantoea*	*agglomerans*	ND	ND	ND
	*Escherichia*	*vulneris*	0.012%	0.004%	ND
CLASS C	*Escherichia*	*coli*	ND	ND	ND
	*Bacillus spp*.		**0.601%**	0.025%	**0.656%**
	*Bacillus*	*cereus*	**0.103%**	ND	ND
	*Clostridium spp*.		**0.190%**	**0.089%**	**1.122%**
	*Clostridium*	*botulinum*	ND	ND	ND
	*Clostridium*	*difficile*	ND	ND	ND
	*Clostridium*	*perfringens*	ND	ND	ND
	*Listeria*	*monocytogenes*	0.015%	0.001%	0.019%
	*Shigella*		ND	ND	ND
	*Staphylococcus*	*aureus*	**0.769%**	0.013%	**1.493%**
	*Yersinia*	*perstis*	**0.118%**	0.023%	ND

*The values are the percentage distribution of the specified genus or species (rows) detected in the corresponding care zone (columns). The distribution values of greater than 0.05% (considered as significant presence of the specified genus/species) are shown in bold (Data from sampling in May 2015). ND: Not detected*.

In summary, the results showed that the physical segregation of a production unit into different care zones has a positive impact on reducing the microbial load within a PIF production unit. However, the reduction in total cell count did not lead to a reduction in either the total viable count or the human associated pathogenic bacterial species. Therefore, better control measures such as stricter monitoring of staff and personal hygiene policies might be necessary to achieve a significant reduction in the human-associated microorganisms in high care. The results also demonstrated that combining the FCM and 16S rDNA sequencing data could be used successfully for hygiene monitoring in PIF production units.

## Author contributions

All authors contributed to the design of the sampling protocol, AA and YC collected samples for FCM and 16S rDNA sequencing studies, respectively, prepared the samples, analyzed the data and drafted the manuscript. AA integrated the FCM and sequencing data, wrote the statistical analysis plan and analyzed the integrated data. SS contributed to the development of sample preparation protocol and analyzed the sequencing data. SF and KJ initiated the study, obtained the funding, designed and supervised the collaborative project, monitored the data collection and contributed to the completion of the manuscript. AA and YC made an equal contribution (co first authors).

## Funding

This work was funded by the Irish Department of Agriculture, Food and the Marine, Ireland, under the Food Institutional Research Measure (FIRM) initiative (project Smart-PIF; reference number 13/F/423).

### Conflict of interest statement

The authors declare that the research was conducted in the absence of any commercial or financial relationships that could be construed as a potential conflict of interest.
