# Supplementary material for: Flow Cytometric and 16S Sequencing Methodologies for Monitoring the Physiological Status of the Microbiome in Powdered Infant Formula Production
Source: Front Microbiol. 2016 Jun 22;7:968. doi: 10.3389/fmicb.2016.00968 (PMC4916166; doi:10.3389/fmicb.2016.00968)
Supplement: Supplementary file 1 [file DataSheet1.pdf]

Supplementary Material

Monitoring the Physiological Status of the Microbiome in Powdered Infant Formula Production Units Using Flow Cytometric, Plating and 16S Sequencing Techniques

Amir H. P. Anvarian†, Yu Cao†, Shabarinath Srikumar, Seamus Fanning, Kieran Jordan*

*** Correspondence:** Kieran Jordan: Kieran.jordan@teagasc.ie

Supplementary Materials — ***Page(s)***

Supplementary Data 1: Fluorophores' stock and working solutions **2-4**
Supplementary Data 2: Gating strategy **5**
Supplementary Table 1: The list of sampling points in PIF production facility. **6**
Supplementary Table 2: Control samples used for performing the colour compensation. **6**
Supplementary Table 3: The colour compensation values used in this study. **7**
Supplementary Table 4: The effects of plating condition on recovery of stressed cells. **8**
Supplementary Table 5: The list of genera identified in each care zone and their contribution to the microbiome. **9-14**
Supplementary Figure 1: Calculating the flow rate of the flow cytometer. **15**

Supplementary Data 1: Fluorophores' stock and working solutions.

Fluorophores

SYTO62: As it was mentioned above, SYTO62 cell-permeant nucleic acid stain was used for discriminating between the cells of interest and the debris. Working solution of SYTO62 (5 µM) was prepared by first warming a vial of the stock solution (5 mM; Molecular Probes, S11344, USA) and bringing it to room temperature. It was then briefly centrifuged (short-spin of 2-3 s) in order to deposit the dimethyl sulphoxide (DMSO) which could interfere with the staining of the cells. Of the stock solution, 10 µL was added to 9,990 µL of filter-sterilized Tris-HCl-EDTA solution (50 mM Tris-HCl and 1 mM EDTA). The latter was prepared by dissolving 6.057 g of Trizma® base (Tris; Sigma-Aldrich 93349) in 800 mL of deH_2O followed by addition of 10 mL of 100 mM Ethylenediaminetetraacetic acid (EDTA). The pH of the solution was then adjusted to 7.5 with 1 M HCl (88.33 mL/L of 35% HCl; VWR 20246.298, Ireland). Finally the volume was adjusted to 1 L. The working solution of SYTO62 was divided into aliquots of 1 mL and stored at -18 °C until use. EDTA is a chelating agent and a scavenger of metal ions. It facilitates the permeabilisation of the outer membrane of the cells, particularly the Gram negative cells by removing the excess extracellular Mg^{2+} and Ca^{2+} as well as reducing the interaction between the lipopolysaccharide (LPS) molecules of the membrane. EDTA solution (100 mM) was prepared by dissolving 14.612 g of EDTA (Sigma ED, USA) in 400 mL of deH_2O with vigorous mixing, adjusting the pH with 10 N NaOH (10 M) to 8.0 and adjusting the volume to 500 mL. It was then filter sterilized to remove the particulates and autoclaved at 121 °C for 15 min.

PI: The cell-impermeant PI is the most commonly used dye for determining membrane integrity, hence viability of the cells. It binds to the DNA of cells that have lost their membrane integrity (dead cells) and is generally excluded from cells with an intact membrane (viable). The working solution of PI was prepared by dissolving 4 mg of PI powder (Sigma P4170;) in 20 mL of deH_2O (299.23 µM). The solution was filter-sterilized using 0.22 µm syringe filters, divided into 1 mL aliquots and stored at 4 °C until use.

BOX: The potentiometric anionic dye bis-(1,3-dibutylbarbituric acid)trimethine oxonol [DiBAC4(3)], also known as bis-oxonol or BOX was used in this study for detecting cells with depolarized membrane (i.e. injured or dead). In this study, BOX was used in combination with PI (i.e. triple-staining with BOX, PI and SYTO62) in order to identify three distinct subpopulations of healthy (PI^-/BOX^- negative; intact membrane integrity and membrane potential), injured (PI^-/BOX^+; intact membrane but collapsed membrane potential) and dead (PI^+/BOX^+; compromised membrane and collapsed membrane potential) cells by plotting green and red fluorescence parameters against each other. BOX stock solution (19.36 mM) was prepared by dissolving 25 mg of BOX (Sigma D8189; USA) in 2.5 mL DMSO (Sigma D8418; USA). It was then divided into 100 µL aliquots and stored at -20 °C until use. Stock solutions were not filter-sterilized. In order to prepare the working solution (19.36 µM), the stock solution was defrosted, 5 µL was added to 4,795 µL of filter-sterilized PBS and supplemented with 200 µL of filter-sterilized 100 mM EDTA (described above). Working solutions were stored for up to a week at 4 °C or at -20 °C until use.

TO and SYTO9: In order to further investigate the viability status of the cells, samples were also dual-stained with PI and either of SYTO9 or its parent compound thiazole orange (TO). Cell-permeable green fluorescent dyes of SYTO9 and TO stain all cells regardless of their physiological state, to varying degrees. However, when used with PI the competition between PI with SYTO9 or TO for DNA binding sites in dead cells and the subsequent displacement of the latters with PI reduces the green fluorescence intensity of the dead cells. Consequently, this leads to identification of two distinct fluorescent subpopulations of green (TO^{+}/PI^{-} or $SYTO9^{+}/PI^{-}$; viable) and red (TO^{-}/PI^{+} or $SYTO9^{-}/PI^{+}$; dead) when the green and red fluorescent parameters are plotted against each other. Working solution of TO (42 μM) was obtained from BD Biosciences (Cell Viability Kit 349483, BD Biosciences; Oxford, UK). Working solution of SYTO9 (250 μM) was prepared by first, warming the vial of stock solution (5 mM; Molecular Probes, S-34854) to room temperature. Stock solution (40 μL) was added to 760 μL DMSO, vortexed and stored at -20 °C for up to a year.

FDA, cFDA and cFDA-SE:

The metabolic activity of the cells within the microbiome was determined based on their esterase activity using FDA and its derivatives cFDA and cFDA-SE. FDA is a non-fluorescent cell permeant esterase substrate which is converted into fluorescent compound of fluorescein upon hydrolysis by intracellular esterases. The presence of carboxyl groups in cFDA and cFDA-SE improves their retention by the cells, making them a useful indicator of membrane integrity as well as esterase activity. Working solution (2.40 mM) of FDA, was prepared on the day of experiment by dissolving 4 mg of FDA (Sigma 7378, USA) in 4 mL of acetone (Fisher Scientific 10131560, Ireland). Working solution of cFDA (2.5 mM) was prepared by dissolving 10 mg of cFDA (Molecular Probes C195, Eugene, USA) in 8,688 μL PBS. Stock solution of cFDA-SE (1.26 μM) was prepared by dissolving 7 mg of cFDA-SE (Sigma 21888, Slovakia) in 10 mL DMSO. Its working solution (25.11 nM) was prepared by diluting the stock solution in filter-sterilized PBS in a 1:50 ratio. Stock and working solutions of cFDA and cFDA-SE were aliquoted into 1 mL portions and stored at -20 °C for up to six months.

SYTOX Green Dead Stain: The cell-impermeant nucleic acid stain of SYTOX Green Dead Cell Stain (Molecular Probes, S-34860) was used as an alternative viability dye to propidium iodide in order to determine the membrane integrity of the cells. SYTOX stock solution (30 μM) was thawed and allowed to equilibrate to room temperature, after which it was diluted in a 1:10 ratio in DMSO (3 μM). The working solutions were divided it ten aliquots of 100 μL (in 0.2 mL microcentrifuge tubes) and stored at -20 °C until use.

Hexidium iodide (HI): HI was used in combination with SYTO9 in order to determine the Gram characteristics of the cells. When bound the to the DNA, the maxima excitation/emission wavelengths for SYTO9 and HI are 485⁄498 nm and 518/600 nm, respectively. Therefore, while both can be excited by the green laser, the emissions of SYTO9 and HI are collected by FL1 and FL3 detectors, respectively. SYTO9 stains most Gram-negative and Gram-positive bacteria whereas HI preferentially stains the Gram-positive cells. When used together, HI displaces the SYTO 9 stain resulting in a decrease in the green fluorescence of Gram-positive cells. Therefore, when green (FL1) and red (FL3) fluorescence are plotted against each other, Gram positive and negative cells could be identified as red and green fluorescent populations, respectively. In order to prepare the stock

solution of HI (10.05 mM), 5 mg of HI powder (Molecular Probes H7593, USA) was dissolved in 1 mL DMSO, divided into 200 µL aliquots and stored at -20 °C until use. For preparing the working solution (25.13 µM). On the day of experiment, the working solution was prepared by diluting the stock solution in PBS in a 1:400 ratio and storing at 4 °C until use.

CTC: CTC was used for studying the respiratory activity of the cells. CTC at its oxidized form is a soluble non fluorescent compound, however, upon its reduction by the electron transfer chain of actively respiring cells, it is converted into non-soluble crystals of red-fluorescent CTC-formazan. The rate of intracellular accumulation of CTC-formazan could be used as a semi-quantitative indicator of the number of healthy and non-healthy cells within the microbial population. Working solution (53.46 mM) of CTC was prepared on the day of experiment by dissolving 5 mg of CTC in 300 µL of ultrapure 0.1 µm filtered water (Sigma, W4502; USA) and stored at 4 °C until use.

Staining buffer

Immediately prior to staining, 250 µL of diluted sample (cell suspension in PBS) was transferred to 12×25 mm flow tubes and supplemented with 20 µL of filter-sterilized 100 mM EDTA and 20 µL of 0.1% (v/v in deH_2O) Polyoxyethylene sorbitan monolaurate (Tween® 20) (Sigma P1379, USA). Therefore, the total volume of an unstained sample (with no added fluorophore) was 290 µL containing 6.90 mM EDTA and 0.007% (v/v) Tween® 20. Tween® 20 was used as a mild non-ionic surfactant for improved permeabilisation of the cell membranes. In order to prepare the 0.1 % (v/v) Tween® 20 solution, 0.1 mL of Tween® 20 was added to 95 mL of deH_2O and the volume adjusted to 100 mL. The solution was then passed through 0.22 µm syringe-filters and stored at 4 °C for up to a week.

Supplementary Data 2: Gating strategy

In summary, the information from P1 was passed through two plots, one representing FSC versus FL5 (plot a[2] and b[2]) and another one SSC versus FL5 (plot a[3] and b[3]). The ranges of FSC and SSC values in plots a(2)/b(2) and a(3)/b(3), respectively, were similar to those in the first plot for the P1 population. By comparing the contour plots of unstained and SYTO 62-stained cells (contour maps of 99% probability), the SYTO 62 positive cells in plots b(2) and b(3) were gated and defined as P2 and P3, respectively. The events present in both P2 and P3 were then passed through two plots, one representing FSC versus FL6 (plots a[4] and b[4]) and the other one SSC versus FL6 (plot a[5] and b[5]) by using the "AND" Boolean logical operator, (i.e. P2 AND P3). FL6 positive populations in plots b(4) and b(5) were gated and defined as P4 and P5, respectively. Using the same Boolean logic, events present in both P4 and P5 gates (i.e. P4 AND P5) of plots b(4) and b(5) were presumed as cells [minus those shown in gate P6 of plot a[6]), plotted in a separate FSC versus SSC-A plot and designated as gate P6. Therefore, any events shown in gate P6 (presumed cells), was also detected in gates P1, P2, P3, P4 and P5. In order to investigate the physiological status of the cells, depending on the number and the type of fluorophores used, the events within P6 were passed through a plot representing either FL1, FL3 or both (FL1 versus FL3).

Supplementary Table 1: The list of sampling points in PIF production facility.

Care zone	Sampling points (number of swabs)
**Low**	- Final product storage area (7)<br>- Two changing rooms (4)*<br>- Corridors (3)<br>- Lorries and forklifts (3)<br>- Packing (2)<br>- Laboratory (1)
**Medium**	- Blending and mixing areas (5)<br>- Two changing rooms (4)*<br>- Evaporation areas (3)<br>- Control rooms (3)<br>- Packaging area (2)<br>- Storage area (2)<br>- Corridor (1)
**High**	- Drying area (7)<br>- Corridors (5)<br>- Two changing rooms (4)*<br>- Blending and mixing area (2)<br>- Control rooms (2)<br>- Packaging area (1)

Notes:
Unless specified otherwise, all samples were taken from floors and drains.
* Two sponges were used for each changing room. One sponge was used for sampling the floor and another one for swabbing one random pair of shoes, one shoe for each side of the sponge.

Supplementary Table 2: Control samples used for performing the colour compensation.

Fluorophore	Detector		Control Sample	
	Primary	Non-primary	Negative	Positive
**SYTO 62**	FL5	FL1, FL3, FL6	Unstained	Alive
**PI**	FL3	FL1, FL5, FL6	Unstained	Dead
**BOX**	FL1	FL3, FL5, FL6	Unstained	Dead
**SYTO 9**	FL1	FL3, FL5, FL6	Unstained	Alive
**TO**	FL1	FL3, FL5, FL6	Unstained	Alive
**FDA**	FL1	FL5, FL6	Unstained	Alive
**cFDA**	FL1	FL3, FL5, FL6	Unstained	Alive
**cFDA-SE**	FL1	FL3, FL5, FL6	Unstained	Alive
**SYTO X Green Dead**	FL1	FL3, FL5, FL6	Unstained	Dead
**HI**	FL3	FL1, FL5, FL6	Unstained	Alive*
**CTC**	FL3	FL5, FL6	Unstained	Alive

Notes: * LGG strain only

Supplementary Table 3: The colour compensation values used in this study.

Fluorophore	Spillover on FL1 (530 ± 30 nm)	Spillover on FL3 (> 670 nm)	Spillover on FL5 (660 ± 20)	Spillover on FL (780 ± 60)
**SYTO 62**	-	23.05%		NC
**PI**	-		1.59%(1); 3.48%(2)	-
**BOX**		0.36%	0.42%	-
**SYTO 9**		0.52%	1.52%	-
**TO**		-	-	-
**FDA**		NA	-	-
**cFDA**		-	-	-
**cFDA-SE**		-	-	-
**SYTO X Green Dead**		-	0.40%	-
**HI**	-		4.13%	-
**CTC**	NA		4.51%	-

Notes: Dash sign "-": Correction not required; NA: Not applicable; NC: Not Corrected. The spillover of the fluorescence of SYTO 62 on FL6 was not corrected in order to utilize this overspill for gating and differentiating between the cells and the debris as previously described. The highlighted cell indicates the primary detector for that fluorophore. (1) When used in combination with SYTO® 9 (PMT voltage of 450 V); (2) When used in combination with other fluorochromes (PMT voltage of 600 nm).

Supplementary Table 4: The effects of plating condition on recovery of stressed cells.

Sample	Growth Medium	Supplement	21 °C		30 °C		37 °C	
			**Incubation Temperature**					
Low Care	NA	None	3.65 ± 0.10	ADG	3.71 ± 0.01	ADG	3.11 ± 0.02	BDG
		Catalase	3.86 ± 0.09	ADEG	3.88 ± 0.06	AEG	2.92 ± 0.29	BDG
		SP	3.98 ± 0.04	AEG	3.97 ± 0.03	AEG	3.09 ± 0.10	BDG
	M9	None	3.82 ± 0.06	ADG	3.81 ± 0.06	ADGH	3.27 ± 0.12	BDG
		Catalase	3.88 ± 0.03	ADG	4.04 ± 0.03	BEH	3.26 ± 0.09	CDG
		SP	3.84 ± 0.02	ADH	3.80 ± 0.04	ADH	3.23 ± 0.07	BDG
	BHI	None	3.62 ± 0.11	ADG	3.80 ± 0.01	ADH	3.09 ± 0.18	BDG
		Catalase	3.83 ± 0.09	ADEG	3.95 ± 0.08	ADGH	3.36 ± 0.18	BDG
		SP	3.89 ± 0.05	AEGH	3.90 ± 0.05	ADGH	3.19 ± 0.03	BDG
Medium Care	NA	None	3.76 ± 0.12	ADG	3.92 ± 0.09	ADG	3.79 ± 0.02	ADG
		Catalase	3.89 ± 0.05	ADG	4.15 ± 0.12	ADG	4.01 ± 0.02	AEG
		SP	3.96 ± 0.09	ADG	4.04 ± 0.09	ADG	3.88 ± 0.02	AFG
	M9	None	3.84 ± 0.13	ADG	4.02 ± 0.07	ADG	3.95 ± 0.04	ADH
		Catalase	3.91 ± 0.14	ABDG	4.18 ± 0.01	AEG	4.01 ± 0.02	BDG
		SP	4.08 ± 0.08	ADG	4.08 ± 0.08	ADEG	4.01 ± 0.07	ADG
	BHI	None	3.90 ± 0.06	ADG	3.96 ± 0.07	ADG	3.72 ± 0.36	ADEGH
		Catalase	3.88 ± 0.00	ADG	4.16 ± 0.01	BEG	4.07 ± 0.05	BDG
		SP	4.14 ± 0.03	AEG	4.06 ± 0.06	ADEG	3.88 ± 0.02	BEG
	NA	None	3.81 ± 0.02	ADG	3.84 ± 0.07	ADFG	3.81 ± 0.02	ADFG
		Catalase	4.11 ± 0.02	ABEG	4.33 ± 0.11	AEG	4.05 ± 0.05	BEG
		SP	3.86 ± 0.00	AFG	3.90 ± 0.05	ADFGH	3.81 ± 0.03	ADFG
	M9	None	3.86 ± 0.05	ADFG	3.91 ± 0.20	ADGH	3.83 ± 0.10	ADGH
		Catalase	4.15 ± 0.03	AEG	4.21 ± 0.16	ADG	4.06 ± 0.09	ADG
		SP	3.91 ± 0.02	ADFH	4.14 ± 0.12	ADG	3.96 ± 0.11	ADG
	BHI	None	3.99 ± 0.00	ADH	4.14 ± 0.04	BDH	3.91 ± 0.04	CDH
		Catalase	4.11 ± 0.03	AEG	3.96 ± 0.13	ABDEG	3.97 ± 0.04	BDG
		SP	3.95 ± 0.00	AFI	3.72 ± 0.13	AEH	3.88 ± 0.08	ADG

Notes: Samples were spread plated in duplicate on NA, M9 or BHI solid growth media (with or without 2000 units per plate catalase or 0.03% sodium pyruvate (SP)) and incubated at either of 21 °C, 30 °C or 37 °C. Data are the calculated mean ± SD log_{10} CFU per cm^2 for two technical duplicate plates of the same sample. Unpaired Student's *t*-test was performed in order to determine the effects of the change in a single parameter (incubation temperature, growth media or supplementation) on the aerobic plate count, when the other two parameters were constant. In each row, data with similar letters of A-C (comparing the effects of incubation temperature only on plate count) are not statistically significant ($p > 0.05$). Similarly, in each column and for each sample, data with similar letters of D-F (comparing the effects of supplementation only, i.e. similar incubation temperature and growth media) and G-I (comparing the effects of growth media only, i.e. similar incubation temperature and supplementation treatment) are not statistically significant ($p > 0.05$) (Data from sampling in May 2015)

Supplementary Table 5: The list of genera identified in each care zone and their contribution to the microbiome.

		Low Care			Medium Care			High Care				
Overall Rank	Genus	Rank	Distribution (%)	Cells/cm^2	Rank	Distribution (%)	Cells/cm^2	Rank	Distribution (%)	Cells/cm^2	Sum	Mean Cells/cm^2
1	Acinetobacter	2	5.640	670	1	27.758	991	5	2.899	136	1797	599
2	Streptococcus	4	4.813	572	14	0.675	24	1	24.882	1163	1759	586
3	Pseudomonas	1	6.601	785	2	13.015	464	3	4.820	225	1475	492
4	Spirosoma	3	5.391	641	70	0.081	3	13	1.107	52	696	232
5	Sphingomonas	5	4.692	558	36	0.162	6	8	1.755	82	646	215
6	Lactococcus	11	1.473	175	8	2.025	72	2	6.963	326	573	191
7	Pedobacter	6	3.388	403	32	0.177	6				409	136
8	Chryseobacterium	12	1.292	154	4	6.676	238	58	0.195	9	401	134
9	Calothrix	8	2.380	283				7	2.007	94	377	126
10	Flavobacterium	7	2.558	304	19	0.486	17	21	0.709	33	355	118
11	Janthinobacterium	9	2.197	261	9	1.830	65	61	0.184	9	335	112
12	Enterobacter	75	0.173	21	3	7.022	251	16	0.978	46	317	106
13	Psychrobacter	16	0.904	107	5	4.294	153	19	0.813	38	299	100
14	Corynebacterium	20	0.752	89	38	0.156	6	4	4.260	199	294	98
15	Hymenobacter	10	1.830	218				50	0.242	11	229	76
16	Lactobacillus	25	0.698	83	37	0.161	6	6	2.823	132	221	74
17	Bacteroides	13	1.247	148	41	0.146	5	11	1.181	55	209	70
18	Paucibacter	33	0.359	43	6	3.193	114	56	0.197	9	166	55
19	Staphylococcus	19	0.769	91				9	1.493	70	161	54
20	Oxalobacter	15	1.173	139	30	0.185	7	48	0.249	12	158	53
21	Roseomonas	14	1.219	145	83	0.054	2	69	0.161	8	154	51
22	Arthrobacter	21	0.731	87	12	0.922	33				120	40
23	Sejongia	17	0.833	99	75	0.075	3	32	0.373	17	119	40
24	Kocuria	32	0.379	45	26	0.257	9	10	1.248	58	113	38
25	Stenotrophomonas	26	0.665	79	31	0.184	7	26	0.450	21	107	36
26	Bacillus	27	0.601	71				23	0.656	31	102	34
27	Dyadobacter	23	0.710	84	23	0.367	13	111	0.055	3	100	33
28	Novosphingobium	18	0.771	92	51	0.122	4				96	32
29	Tolumonas	36	0.324	39	11	1.022	36	41	0.286	13	88	29
30	Variovorax	24	0.709	84	72	0.078	3				87	29
31	Acidisoma	22	0.716	85							85	28
32	Enhydrobacter	79	0.163	19	10	1.234	44	28	0.417	19	83	28
33	Clostridium	68	0.190	23	67	0.089	3	12	1.122	52	78	26
34	Vagococcus	52	0.253	30	28	0.227	8	18	0.834	39	77	26
35	Leuconostoc	28	0.486	58	35	0.174	6	42	0.271	13	77	26
36	Meiothermus				7	2.115	75				75	25
37	Paracoccus	39	0.316	38	17	0.565	20	39	0.331	15	73	24
38	Blautia	73	0.182	22				14	1.090	51	73	24
39	Mycobacterium	30	0.418	50				31	0.385	18	68	23

40	Enterococcus	49	0.258	31	27	0.255	9	25	0.529	25	65	22
41	Methylobacterium	29	0.441	52				45	0.255	12	64	21
42	Micrococcus	80	0.156	19	65	0.091	3	20	0.777	36	58	19
43	Prevotella							15	1.082	51	51	17
44	Sphingobacterium	40	0.307	36	22	0.387	14				50	17
45	Runella	63	0.196	23	13	0.746	27				50	17
46	Rubellimicrobium	38	0.319	38				55	0.204	10	47	16
47	Kaistobacter	31	0.398	47							47	16
48	Devosia	34	0.342	41				98	0.079	4	44	15
49	Faecalibacterium							17	0.930	43	43	14
50	Luteolibacter	62	0.201	24	77	0.070	2	34	0.365	17	43	14
51	Delftia	167	0.060	7	18	0.565	20	37	0.343	16	43	14
52	Dolichospermum	47	0.268	32				51	0.232	11	43	14
53	Chitinophaga	35	0.329	39	68	0.086	3				42	14
54	Carnobacterium	45	0.284	34				66	0.166	8	42	14
55	Xanthomonas				15	0.660	24	33	0.370	17	41	14
56	Microcoleus	51	0.256	30				57	0.196	9	40	13
57	Rhodococcus	48	0.265	32	62	0.097	3	94	0.087	4	39	13
58	Megasphaera	53	0.241	29	25	0.277	10				39	13
59	Nostoc	66	0.191	23				38	0.335	16	38	13
60	Rickettsia	37	0.319	38							38	13
61	Bradyrhizobium	43	0.287	34	60	0.099	4				38	13
62	Agrobacterium	54	0.228	27				52	0.222	10	37	12
63	Sphingobium	42	0.294	35				115	0.052	2	37	12
64	Deinococcus	41	0.295	35							35	12
65	Alkanindiges	44	0.285	34							34	11
66	Nocardioides	50	0.257	31	71	0.080	3				33	11
67	Leptolyngbya	70	0.189	22				54	0.221	10	33	11
68	Jeotgalicoccus	46	0.273	32							32	11
69	Bifidobacterium	119	0.099	12				27	0.430	20	32	11
70	Oscillospira							22	0.676	32	32	11
71	Segetibacter	56	0.221	26	50	0.127	5				31	10
72	Gluconobacter	55	0.226	27	64	0.094	3				30	10
73	Brevundimonas	74	0.182	22	73	0.078	3	79	0.122	6	30	10
74	Myroides	67	0.190	23	52	0.121	4	118	0.050	2	29	10
75	Chondromyces	61	0.205	24	48	0.128	5				29	10
76	Dokdonella	57	0.220	26				112	0.055	3	29	10
77	Giesbergeria	84	0.149	18	45	0.136	5	75	0.131	6	29	10
78	Comamonas	163	0.063	7	16	0.574	20				28	9
79	Rhodoferax							24	0.595	28	28	9
80	Wautersiella	111	0.110	13	21	0.412	15				28	9
81	Saccharopolyspora	82	0.152	18	66	0.090	3	74	0.133	6	27	9
82	Labrys	58	0.217	26							26	9
83	Gardnerella	59	0.213	25							25	8
84	Cellvibrio	60	0.210	25							25	8
85	Bdellovibrio	76	0.171	20				91	0.094	4	25	8
86	Propionibacterium	174	0.055	7				36	0.355	17	23	8
87	Alkalibacterium	86	0.140	17				73	0.135	6	23	8

88	Polaromonas	88	0.140	17	34	0.175	6				23	8
89	Acidiphilium	64	0.192	23							23	8
90	Burkholderia	65	0.191	23							23	8
91	Streptomyces	102	0.121	14	46	0.135	5	103	0.074	3	23	8
92	Oerskovia	69	0.190	23							23	8
93	Granulicella	71	0.187	22							22	7
94	Luteimonas				40	0.146	5	35	0.359	17	22	7
95	Peptoniphilus	93	0.132	16				76	0.131	6	22	7
96	Campylobacter	72	0.183	22							22	7
97	Geobacillus	107	0.112	13	54	0.113	4	95	0.087	4	21	7
98	Aerococcus	135	0.081	10				47	0.249	12	21	7
99	Chthoniobacter	91	0.134	16	47	0.133	5				21	7
100	Heliorestis	146	0.070	8				44	0.256	12	20	7
101	Thalassospira	77	0.170	20							20	7
102	Actinomyces	78	0.169	20							20	7
103	Erwinia	89	0.137	16				99	0.076	4	20	7
104	Brachybacterium	110	0.110	13	87	0.051	2	84	0.104	5	20	7
105	Acidovorax	101	0.122	15	39	0.147	5				20	7
106	Anaerococcus	109	0.110	13				77	0.129	6	19	6
107	Lysobacter	98	0.126	15				93	0.088	4	19	6
108	Macrococcus	131	0.087	10	58	0.102	4	81	0.109	5	19	6
109	Dialister							29	0.402	19	19	6
110	Anoxybacillus							30	0.391	18	18	6
111	Methylotenera	81	0.153	18							18	6
112	Singulisphaera	83	0.151	18							18	6
113	Rathayibacter	85	0.146	17							17	6
114	Halomonas	143	0.073	9	76	0.071	3	78	0.124	6	17	6
115	Polynucleobacter	97	0.127	15	89	0.051	2				17	6
116	Alkaliphilus	118	0.099	12				83	0.107	5	17	6
117	Polaribacter	87	0.140	17							17	6
118	Paenibacillus	161	0.064	8				59	0.190	9	16	5
119	Microbacterium	112	0.109	13				102	0.074	3	16	5
120	Serratia	90	0.136	16							16	5
121	Thermomonas	139	0.079	9	79	0.058	2	87	0.098	5	16	5
122	Chroococcidiopsis	92	0.133	16							16	5
123	Curtobacterium	94	0.131	16							16	5
124	Nesterenkonia	158	0.065	8				67	0.165	8	15	5
125	Frankia	95	0.129	15							15	5
126	Arcobacter				20	0.427	15				15	5
127	Arthronema	96	0.127	15							15	5
128	Luteibacter	99	0.125	15							15	5
129	Xenophilus	100	0.124	15							15	5
130	Desulfovibrio	114	0.106	13	81	0.056	2				15	5
131	Legionella	134	0.082	10	49	0.127	5				14	5
132	Olivibacter	103	0.120	14							14	5
133	Yersinia	104	0.118	14							14	5
134	Sanguibacter	105	0.118	14							14	5
135	Demequina	106	0.115	14							14	5

136	Brevibacterium							40	0.289	**14**	14	**5**
137	Curvibacter	123	0.093	**11**				117	0.050	**2**	13	**4**
138	Moraxella				44	0.141	**5**	62	0.177	**8**	13	**4**
139	Schlegelella	108	0.111	**13**							13	**4**
140	Asticcacaulis	113	0.107	**13**							13	**4**
141	Skermanella	155	0.066	**8**				89	0.096	**4**	12	**4**
142	Rothia							43	0.262	**12**	12	**4**
143	Cellulomonas	115	0.103	**12**							12	**4**
144	Aurantimonas	116	0.102	**12**							12	**4**
145	Azohydromonas	148	0.069	**8**	57	0.107	**4**				12	**4**
146	Phycicoccus	117	0.101	**12**							12	**4**
147	Brevibacillus							46	0.253	**12**	12	**4**
148	Prosthecobacter	120	0.097	**12**							12	**4**
149	Cystobacter							49	0.246	**12**	12	**4**
150	Ancylobacter	121	0.096	**11**							11	**4**
151	Wohlfahrtiimonas	159	0.065	**8**	59	0.100	**4**				11	**4**
152	Rickettsiella	122	0.094	**11**							11	**4**
153	Desemzia	124	0.093	**11**							11	**4**
154	Pediococcus	173	0.055	**7**				90	0.095	**4**	11	**4**
155	Methylosinus	125	0.092	**11**							11	**4**
156	Microcystis	126	0.092	**11**							11	**4**
157	Flavisolibacter	127	0.091	**11**							11	**4**
158	Rhodobacter	128	0.091	**11**							11	**4**
159	Maricaulis	129	0.090	**11**							11	**4**
160	Gillisia	130	0.088	**10**							10	3
161	Klebsiella				24	0.291	**10**				10	3
162	Pseudoxanthomonas							53	0.222	**10**	10	3
163	Salinibacterium	132	0.084	**10**							10	3
164	Marinitoga	133	0.083	**10**							10	3
165	Tepidimonas	178	0.052	6	61	0.099	**4**				10	3
166	Thermogemmatisp	136	0.081	**10**							10	3
167	Blastococcus	137	0.079	9							9	3
168	Rhodocyclus	138	0.079	9							9	3
169	Emticicia	140	0.078	9							9	3
170	Actinomadura	141	0.077	9							9	3
171	Acidisphaera	142	0.075	9							9	3
172	Porphyromonas							60	0.188	9	9	3
173	Oscillatoria	144	0.072	9							9	3
174	Fulvivirga	145	0.070	8							8	3
175	Mitsuokella							63	0.176	8	8	3
176	Leucobacter	147	0.069	8							8	3
177	Phormidium							64	0.175	8	8	3
178	Geodermatophilus	149	0.068	8							8	3
179	Lautropia	150	0.068	8							8	3
180	Pseudoalteromona	151	0.068	8							8	3
181	Sporosarcina	152	0.068	8							8	3
182	Zymomonas	153	0.068	8							8	3
183	Luteococcus							65	0.171	8	8	3

184 Mycoplasma	154	0.066	8							8	3
185 Terriglobus	156	0.066	8							8	3
186 Agrococcus	157	0.065	8							8	3
187 Uliginosibacterium	160	0.064	8							8	3
188 Prauserella							68	0.162	8	8	3
189 Chroococcus	162	0.063	7							7	2
190 Pseudaminobacter	164	0.063	7							7	2
191 Bergeyella	165	0.062	7							7	2
192 Rhodoplanes	166	0.062	7							7	2
193 Alishewanella							70	0.154	7	7	2
194 Hydrogenophilus				29	0.201	7				7	2
195 Roseococcus	168	0.060	7							7	2
196 Williamsia	169	0.059	7							7	2
197 Citricoccus	170	0.058	7							7	2
198 Modestobacter	171	0.058	7							7	2
199 Thermobacillus							71	0.140	7	7	2
200 Lutibacterium	172	0.055	7							7	2
201 Bosea	175	0.054	6							6	2
202 Conexibacter	176	0.054	6							6	2
203 Trichococcus	177	0.054	6							6	2
204 Granulicatella							72	0.137	6	6	2
205 Providencia				33	0.176	6				6	2
206 Haliangium	179	0.051	6							6	2
207 Rhodanobacter	180	0.050	6							6	2
208 Isoptericola	181	0.050	6							6	2
209 Arenimonas				86	0.051	2	105	0.073	3	5	2
210 Ferrimonas				42	0.146	5				5	2
211 Thermus				43	0.143	5				5	2
212 Erysipelothrix							80	0.109	5	5	2
213 Sutterella							82	0.109	5	5	2
214 Butyrivibrio							85	0.103	5	5	2
215 Mechercharimyces							86	0.101	5	5	2
216 Eubacterium							88	0.097	5	5	2
217 Desulfonatronum							92	0.093	4	4	1
218 Citrobacter				53	0.116	4				4	1
219 Facklamia							96	0.085	4	4	1
220 Mannheimia							97	0.084	4	4	1
221 Candidatus Scalindua				55	0.109	4				4	1
222 Niastella				56	0.109	4				4	1
223 Hydrogenophaga							100	0.075	4	4	1
224 Mycoplana							101	0.075	4	4	1
225 Tetragenococcus							104	0.074	3	3	1
226 Arthrospira				63	0.095	3				3	1
227 Yaniella							106	0.070	3	3	1
228 Amycolatopsis							107	0.069	3	3	1
229 Gemmata				69	0.085	3				3	1
230 Actinobaculum							108	0.063	3	3	1
231 Azoarcus							109	0.063	3	3	1

232 Desulfonauticus				110	0.060	3	3	1
233 Steroidobacter	74	0.075	3				3	1
234 Candidatus Endobugula				113	0.053	2	2	1
235 Alloiococcus				114	0.052	2	2	1
236 Vibrio				116	0.051	2	2	1
237 Sharpea				119	0.050	2	2	1
238 Lewinella	78	0.065	2				2	1
239 Vogesella	80	0.057	2				2	1
240 Shewanella	82	0.055	2				2	1
241 Oenococcus	84	0.054	2				2	1
242 Marinomonas	85	0.051	2				2	1
243 Plesiomonas	88	0.051	2				2	1

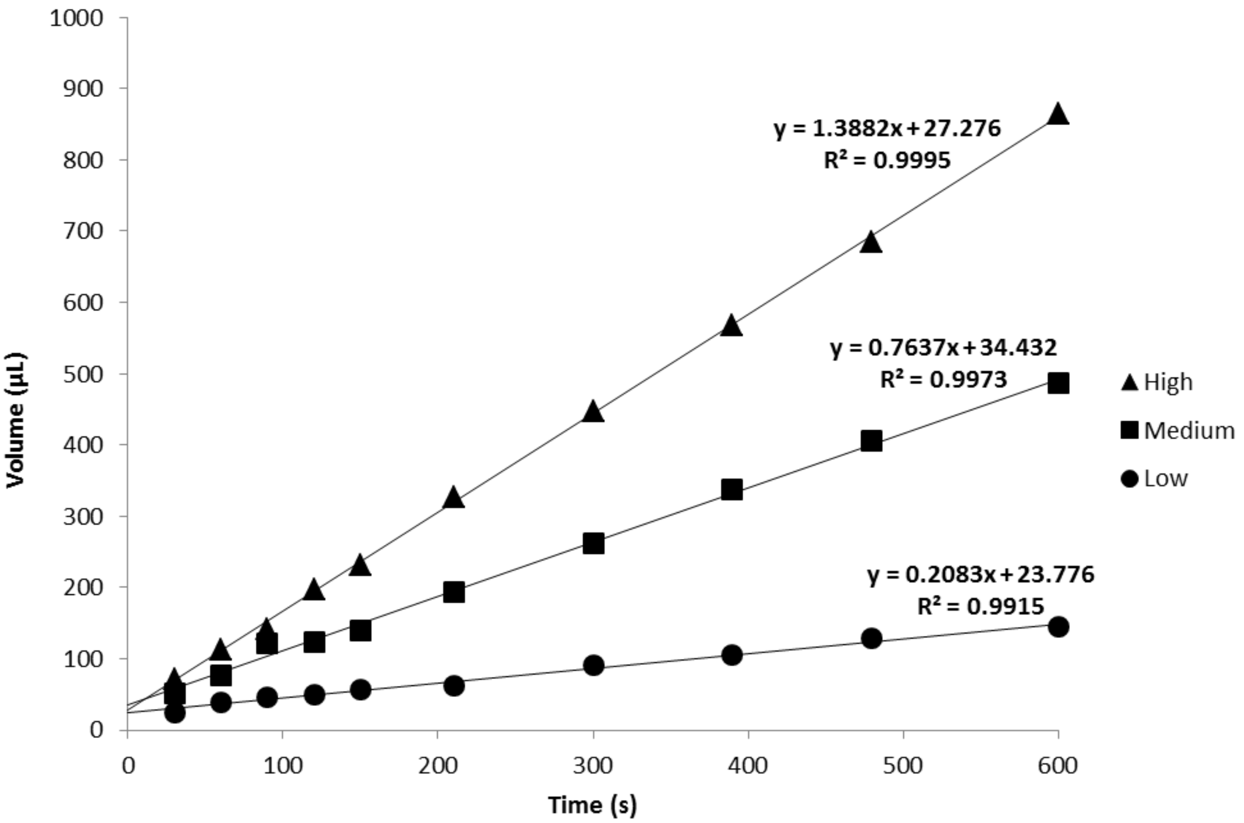

Supplementary Figure 1: Calculating the flow rate of the flow cytometer. The slope of the trendline's linear equation was multiplied by the acquisition time to determine the volume of sample analyzed. The intercept of the equation is indicative of the sample volume that was retained by the instrument (so-called dead volume) but not analyzed.
